# The human cytomegalovirus decathlon: Ten critical replication events provide opportunities for restriction

**DOI:** 10.3389/fcell.2022.1053139

**Published:** 2022-11-25

**Authors:** Declan L. Turner, Rommel A. Mathias

**Affiliations:** ^1^ Department of Microbiology, Infection and Immunity Program, Monash Biomedicine Discovery Institute, Monash University, Melbourne, VIC, Australia; ^2^ Department of Biochemistry and Molecular Biology, Monash University, Melbourne, VIC, Australia

**Keywords:** viral replication, antiviral therapeutic, herpes viral infection, HCMV (human cytomegalovirus), virion assembly

## Abstract

Human cytomegalovirus (HCMV) is a ubiquitous human pathogen that can cause severe disease in immunocompromised individuals, transplant recipients, and to the developing foetus during pregnancy. There is no protective vaccine currently available, and with only a limited number of antiviral drug options, resistant strains are constantly emerging. Successful completion of HCMV replication is an elegant feat from a molecular perspective, with both host and viral processes required at various stages. Remarkably, HCMV and other herpesviruses have protracted replication cycles, large genomes, complex virion structure and complicated nuclear and cytoplasmic replication events. In this review, we outline the 10 essential stages the virus must navigate to successfully complete replication. As each individual event along the replication continuum poses as a potential barrier for restriction, these essential checkpoints represent potential targets for antiviral development.

## Introduction

HCMV is a widespread human pathogen with highest infection rates in low socioeconomic demographics, and seropositivity greater than 90 percent in some populations (Cannon et al., 2010). HCMV establishes a lifelong latent infection, with periodic shedding of the virus providing a persistent source of transmission (Goodrum et al., 2012). Primary infection in healthy individuals is often asymptomatic or accompanied by symptoms of mild mononucleosis, fever, and sore throat. However, in rare cases, severe complications including viral hepatitis, colitis, splenomegaly, and encephalitis can occur (Horwitz et al., 1986; Rafailidis et al., 2008). For the immunocompromised, including transplant recipients, HIV positive individuals or those receiving cancer chemotherapy, HCMV infection can cause serious complications as is illustrated by 90 percent of AIDS patients showing HCMV cytopathology during autopsy (Reichert et al., 1983). Solid organ and hematopoietic stem cell transplant (HSCT) recipients are also at elevated risk of HCMV disease due to the immunosuppressive drug regimen required to stop transplant rejection and graft-versus-host disease (Razonable, 2010). The risks are further elevated for HCMV negative recipients receiving HCMV positive tissue with a bidirectional correlation between HCMV infection and transplant rejection (Razonable et al., 2001). For HCMV negative organs transplanted to a positive recipient, the inflammatory environment can reactivate the latent virus, while in the opposite situation, the immunosuppressive drugs compromise a robust immune response (Ramanan and Razonable, 2013).

HCMV is also the leading cause of birth defects due to an infectious agent, which results from intrauterine transmission of the virus to the developing foetus during productive infection of the mother (Bonalumi et al., 2011; Manicklal et al., 2013). HCMV affects approximately 1 in 200 pregnancies (Manicklal et al., 2013), with a transmission rate to the foetus of approximately 30% during primary infection and 1.2% for reactivation of a latent infection (Kenneson and Cannon, 2007). Approximately 10% of congenitally infected infants will show symptoms at birth, the most common being sensorineural hearing and vision impairments which affect 3.5% of infants infected (Grosse et al., 2008; Manicklal et al., 2013). Additionally, mild to severe learning disabilities, failure to thrive, microcephaly or still birth can occur in the most severe cases.

The frontline drugs for treatment of HCMV infection and prophylaxis in organ and HSCT recipients are the acyclovir derivatives ganciclovir and its oral prodrug valganciclovir (Acosta et al., 2020). They are both synthetic analogues of 2′-deoxy-guanosine which are selectively phosphorylated by the viral kinase UL97 in infected cells (Littler et al., 1992). Host cell kinases provide further phosphorylation to the triphosphate form which has strong affinity for UL54, the viral DNA polymerase, which results in chain termination once incorporated into the daughter strand (King, 1988; Matthews and Boehme, 1988). Foscarnet, a pyrophosphate analogue, and cidofovir, a monophosphate nucleotide analogue, also have increased affinity for UL54 and block DNA replication through distinct mechanisms (Ahmed, 2011). Both are reserved as second-line treatments due to considerable toxicity. Maribavir blocks the action of the UL97 kinase (Prichard, 2009) and has recently completed a phase III trial where it achieved the primary endpoints (Avery et al., 2021). Letermovir is a specific inhibitor of the terminase complex and was recently licensed for HCMV prophylaxis in HSCT recipients (El Helou and Razonable, 2019). Maribavir and letermovir are the first HCMV drugs which do not target UL54, however, maribavir likely blocks phosphorylation of ganciclovir through UL97 inhibition which may exclude combination treatment (Acosta et al., 2020). Monotherapy readily selects for resistant mutants, particularly given the low genetic barrier to resistance and chronic persistence of HCMV infection in immunocompromised patients (Chou et al., 2018; Razonable, 2018; Paolucci et al., 2021). Drug resistant mutants of all licensed drugs have already emerged, including letermovir, and highlights the need for new drugs, vaccines and therapeutic strategies (Eid et al., 2008; Rolling, 2017; Razonable, 2018). Combination therapy with a cocktail of compatible antivirals, peptides or antibodies targeting distinct stages of the replication cycle lowers the required dose thus increasing tolerability while also reducing the probability of resistant mutants emerging. Another approach is to drug a host cell protein or pathway which is essential for completion of viral replication. Resistant mutants will not be selected for at any meaningful frequency due to higher genetic barriers to resistance compared to viral targets, however, toxicity is a serious concern and must be carefully balanced for overall clinical benefit (Lin and Gallay, 2013; Ji and Li, 2020; Lingappa et al., 2021). It is for these reasons that the distinct stages of the viral replication cycle are understood and characterised, towards identifying vulnerabilities that can be exploited by novel antivirals.

## Stage 1: Viral entry into host cells

HCMV belongs to the *Betaherpesvirinae* subfamily of viruses which also includes human herpesvirus 6A, 6B and 7. HCMV has a 235 kb (strain dependent) linear double stranded DNA (dsDNA) genome containing more than 170 open reading frames packaged tightly inside a pseudo-icosahedral nucleocapsid, a thick layer of tegument proteins, and host cell derived envelope containing glycoprotein complexes. HCMV can infect almost all cell types which is consistent with the diverse symptoms of HCMV disease. HCMV has a strong tropism for fibroblasts, epithelial, endothelial, smooth muscle and placental cells (Sinzger et al., 2008; Revello and Gerna, 2010).

**FIGURE 1:**
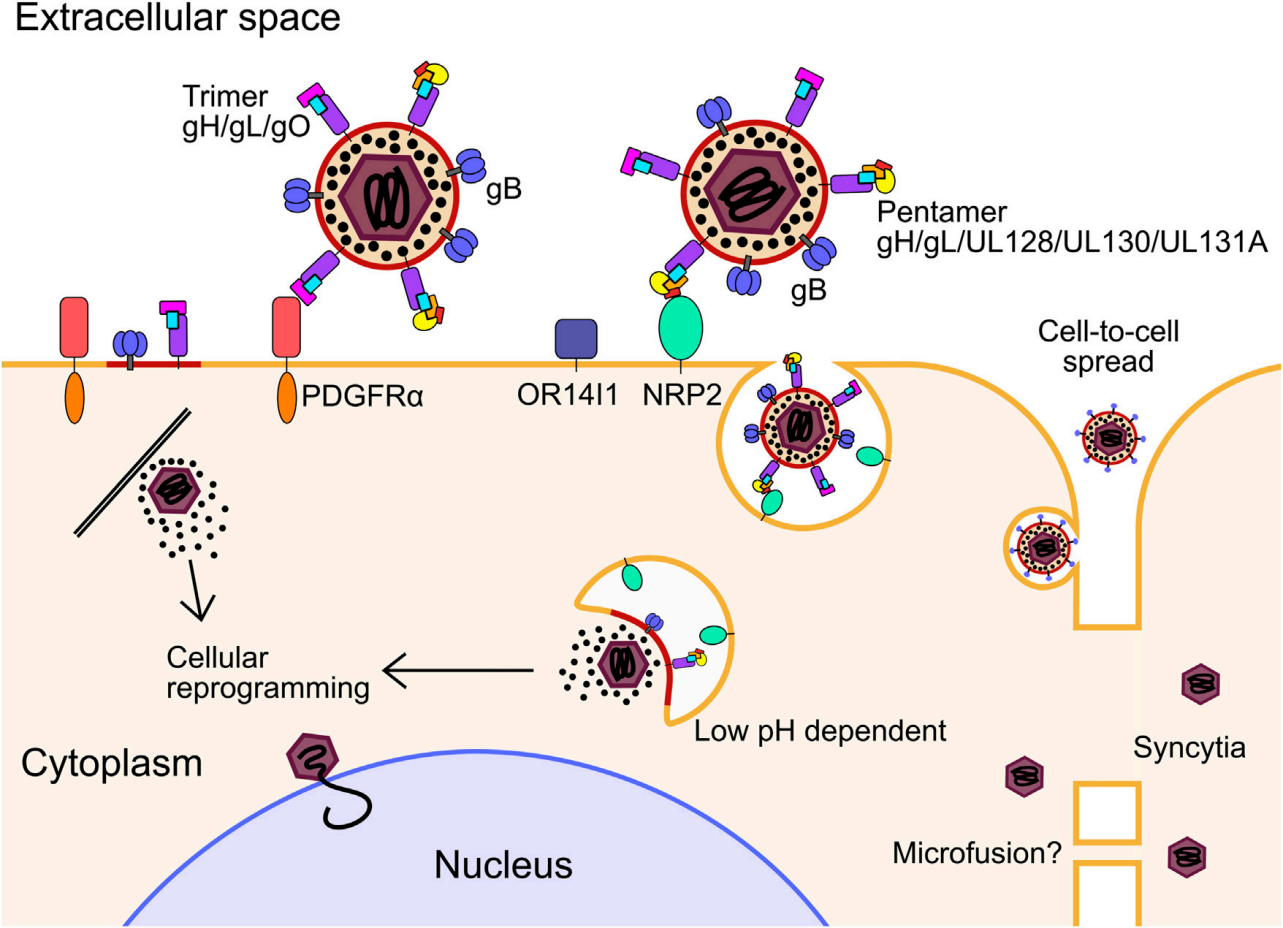
For infection in fibroblasts, the glycoprotein trimer consisting of gH, gL and gO binds to PDGFRα. Fusion between the envelope and cell membrane is mediated by gB and the tegumented nucleocapsid is released into the cytoplasm. For infection of epithelial, endothelial, myeloid, and likely many other cell types, the glycoprotein pentamer consisting of gH, gL, UL128, UL130 and UL131A binds to either OR14I1 or NRP2 on the cell surface and induces endocytosis of the virion. Pentamer mediated entry is dependent on acidification of the endosome as well as gO. The fusion step is mediated by gB to release the tegumented capsid into the cytoplasm, and tegument proteins dissociate and begin to reprogram the host cell. The capsid traffics to the nucleus for genome deposition. PDGFRα, platelet derived growth factor receptor alpha; NRP2, neuropilin; gB, envelope glycoprotein B.

HCMV has multiple envelope glycoprotein complexes that engage unrelated receptors on the cell surface, determining entry of the virion into different cell types. These are primarily the gH/gL/gO trimer and gH/gL/UL128/UL130/UL131A pentamer. HCMV clinical isolates serially passaged in fibroblasts readily accumulate mutations in UL128, UL130 and UL131A, and are dispensable for entry into fibroblasts (Dargan et al., 2010; Stanton et al., 2010), further showing the trimer and pentamer are mutually exclusive complexes (Ciferri et al., 2015). Platelet-derived growth factor receptor α (PDGFRα) is highly abundant on the surface of fibroblasts and was identified as the predominant receptor for the trimer which interacts through contact with gO (Soroceanu et al., 2008; Kabanova et al., 2016; Wu et al., 2017; Wu et al., 2018; Kschonsak et al., 2021), and is independent of the PDGFRα intracellular kinase domain (Wu et al., 2018) (Figure 1). Transforming growth factor beta receptor type III (TGFβR3) functions as a second receptor for trimer, although it appears to not be able to efficiently mediate entry (Martinez-Martin et al., 2018; Kschonsak et al., 2021). Trimer-dependent entry is pH independent and was initially understood to occur by direct fusion on the target cell surface, although more recent work has shown entry to be dynamin II-dependent, and is consistent with rapid macropinocytosis (Vanarsdall et al., 2012; Hetzenecker et al., 2016). Recent high-throughput screens of host cell surface proteins have identified neuropilin 2 (NRP2) (Martinez-Martin et al., 2018), and OR14I1 (E et al., 2019) as predominant entry receptors of the pentamer for epithelial, endothelial and myeloid cell entry, with the structural basis for NRP2 binding by pentamer solved (Wrapp et al., 2022) (Figure 1). In addition to this, various integrins have been identified as potential co-receptors for trimer and pentamer-dependent mechanisms (Feire et al., 2004; Wang et al., 2005; Feire et al., 2010). Pentamer-dependent entry also requires dynamin II (Vanarsdall et al., 2012), but unlike the trimer, requires low pH in the endosome for delivery of the nucleocapsid (Ryckman et al., 2006; Vanarsdall et al., 2012) (Figure 1). Interestingly, overexpression of PDGFRα in epithelial cells and monocytes can rescue susceptibility to pentamer-null virus strains, providing evidence that there is not a cell type specific block in internalisation and membrane fusion, but rather receptor levels remain below a functional threshold (Vanarsdall et al., 2012; Wu et al., 2018). Similarly, restoration of pentamer in the fibroblast passaged strain AD169 rescued robust extracellular virion production in epithelial and endothelial cells (ECs) (Wang and Shenk, 2005; Adler et al., 2006).

It was initially thought that the pentamer alone is sufficient for epithelial cell attachment and entry, while the trimer is sufficient in fibroblasts. However, it has been shown that gO null virus cannot enter epithelial cells, indicating that trimer is essential for entry into all cell types, including when PDGFRα is blocked (Zhou et al., 2015; Kabanova et al., 2016). Recently, functionally important domains of gO have been identified that function after receptor binding (Chin et al., 2022). The authors speculated on several models which included activation of gB for fusion. After receptor binding, when the envelope and target cell membrane are proximal, gB mediates fusion of the membranes, independently of receptor binding (Isaacson and Compton, 2009), which is further evidenced by envelope fusion of gB null virions when gB is expressed in *trans* on the target cell surface (Wille et al., 2013). Whether gB interacts with the trimer to initiate fusion, or solely proximity to the target membrane is sufficient, remains an open question.

In addition to cell-free virions, cell-to-cell HCMV spread can occur in tissue culture monolayers. This is likely to be the main route of dissemination throughout the body, as clinical isolates are highly cell-associated before passage in culture (Sinzger et al., 1999; Dargan et al., 2010). It was found that PDGFRα is required for cell-to-cell spread in fibroblasts for virus strains only expressing trimer, while pentamer expressing virus could spread in PDGFRα knock-out cells (Wu et al., 2018). Pentamer dependent spread is primarily cell-associated, with fewer passages in culture correlating with higher pentamer abundance in virions and greater entry efficiency in epithelial cells (Murrell et al., 2013). Further, pentamer dependent cell-to-cell spread is resistant to antibody neutralisation compared to cell-free spread which further supports this mode of dissemination *in vivo* (Murrell et al., 2017). It must be noted that other studies have observed antibody neutralisation of cell-to-cell spread using different virus strains which have altered ratios of trimer and pentamer in their envelopes, which may explain these differences and potentially offer insights into different mechanisms of spread (Li et al., 2015; Klupp et al., 2017; Reuter et al., 2022). Curiously, virions lacking the assembly tegument protein UL99 were still able to spread cell-to-cell in fibroblasts, despite a defect in virion envelopment, which may indicate a distinct mechanism (Silva et al., 2005). The exact mechanism of cell-to-cell spread for different strains and cell types has not yet been solved explicitly, however, the requirement for functional target cell receptors, and trimer and pentamer glycoproteins, point to a conventional entry mechanism between proximal cells which is resistant to antibody neutralisation at physiological concentrations, at least with the strains and cell types assayed. More exotic mechanisms such as micro fusions between adjacent cell membranes (Gerna et al., 2000) or syncytia (Gerna et al., 2016; Cui et al., 2017) cannot be ruled out in certain experimental or physiological conditions (Figure 1).

## Stage 2: Nuclear trafficking and early viral gene expression

After membrane fusion, the tegumented nucleocapsid is delivered to the cytoplasm and begins trafficking towards the nucleus using the host cell cytoskeleton (Ogawa-Goto et al., 2003; Miller and Hertel, 2009). Concurrently, highly abundant tegument proteins are known to dissociate from the nucleocapsid and begin re-programming the host cell (Kalejta et al., 2008). Tegument proteins have diverse functions which begin with delivery inside virions but also include newly synthesised pools of the molecules during infection. Tegument proteins have multiple functions (beyond the scope of this review) throughout the replication cycle which poses challenges for characterisation (Kalejta, 2008). At the nuclear membrane, the viral genome is understood to be released through the nuclear pore complex (NPC), based on studies of herpes simplex virus 1 (HSV-1) [reviewed (Fay and Panté, 2015)]. Only recently have HCMV specific mechanisms of nuclear entry been investigated, revealing the requirement of Stimulator of interferon genes protein (STING) for genome delivery (Hong et al., 2021).

Once nuclear, the HCMV genome circularises, likely by direct end joining as for HSV-1 (Strang and Stow, 2005), and early viral gene expression commences. HCMV has three traditional kinetic classes of gene expression: immediate early (IE), delayed early (DE) and late (L). IE genes are multifunctional effectors that disrupt antiviral processes and act as transcription factors for DE gene expression. DE genes modulate the cell cycle, nucleotide and lipid metabolism and encode the viral DNA polymerase complex [reviewed (Shenk and Alwine, 2014)]. They also include non-essential immuno-modulatory effectors such as major histocompatibility complex (MHC) class 1, interleukin 10 and fc-gamma receptor mimics among others (Powers et al., 2008; Corrales-Aguilar et al., 2014). L gene products are predominantly virion components such as capsid proteins, tegument proteins and envelope glycoproteins. Recently, a more complex picture of late gene expression has emerged (Stern-Ginossar et al., 2012; Weekes et al., 2014; Rozman et al., 2022), and is further discussed within Stage 5.

**FIGURE 2:**
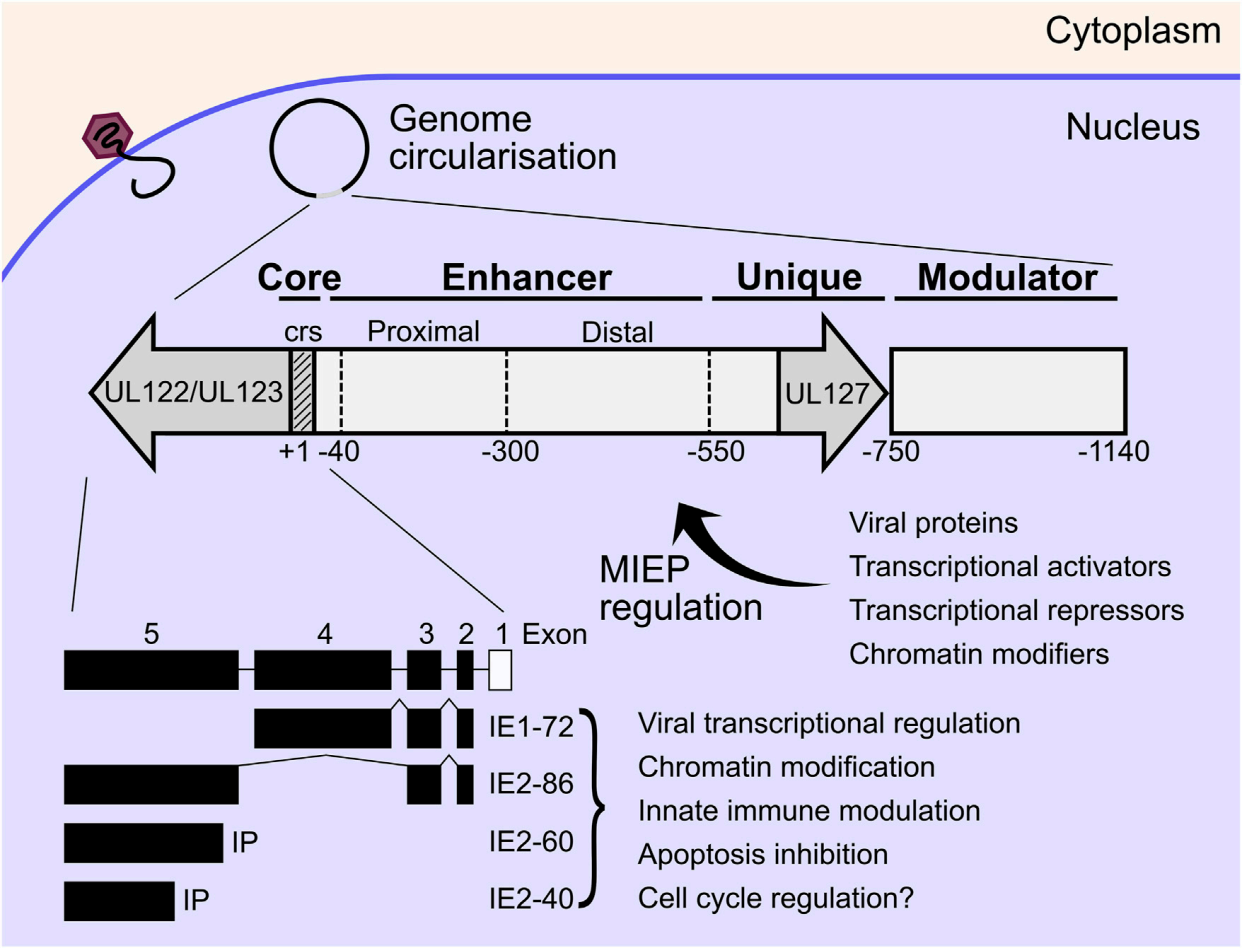
Inside the host cell nucleus, viral genomes circularise, and IE gene expression commences independently of viral protein synthesis. The MIEP is immediately upstream of the IE genes and acts as a hub for transcriptional activation or repression of the IE proteins by diverse host and viral factors. MIEP regulation also dictates the switch from lytic replication to latency and *vice versa*. The MIEP begins immediately upstream of the *UL122/UL123* ORF and consists of the core (+1 to −40), enhancer (−40 to −550), unique (−550 to −750) and modulator (−750 to −1,140) regions. The IE1 and IE2 proteins are the main IE effectors which are encoded by the UL122/UL123 ORF by alternative splicing. IE1-72 and IE2-86 are multifunctional proteins that transactivate DE viral genes, remodel chromatin, disrupt interferon signalling, and inhibit apoptosis to create a conducive cellular environment for viral replication. IE, immediate early; MIEP, major immediate early promoter; ORF, open reading frame; DE, delayed early; crs, *cis*-repression sequence.

The HCMV major immediate early promoter (MIEP) and enhancer region is a master regulator of HCMV IE gene expression, that initiates replication cycle progression without viral protein synthesis (Stinski and Isomura, 2008). The MIEP is the focal point where diverse host and viral signals are integrated to initiate or suppress active replication. The MIEP contains a promoter region (+1 to −40 bp from the transcriptional start site of *IE1/2*), an enhancer region (−40 to −550 bp), a unique region (-550 to -750) and a modulator (−750 to −1,140) [reviewed (Adamson and Nevels, 2020)] (Figure 2). The MIEP is bidirectional, however transcription of the *UL127* open reading frame is inhibited by a repressor sequence in the unique region which is bound by cellular homeobox proteins (Lundquist et al., 1999; Angulo et al., 2000; Chao et al., 2004; Lashmit et al., 2004; Lee et al., 2007). The enhancer and modulator region greatly amplifies transcription and is bound by a plethora of cellular transcription factors, some of which have inhibitory function (Adamson and Nevels, 2020). In addition to transcription factors, herpesviral genomes are chromatinised during lytic and latent infection, which adds another layer of transcriptional regulation of viral gene expression. Post translational modifications of histones including acetylation, methylation and phosphorylation have all been shown to modulate transcriptional activity of the MIEP [reviewed (Paulus et al., 2010; Nevels et al., 2011; Knipe et al., 2013; Adamson and Nevels, 2020)]. Transcriptional activity of the HCMV MIEP is also activated by cellular and immune signalling pathways, and includes the mitogen and stress activated protein kinase (MSK) family acting through cAMP-response element binding protein (CREB) (Kew et al., 2014), tumour necrosis factor α (TNF-α) and nuclear factor κB (NF-κB) (Stein et al., 1993; Döcke et al., 1994; Prösch et al., 1995), as well as reactive oxygen species acting through activator protein 1 (AP-1) complexes (Kim et al., 2005). The MIEP is also inhibited directly and indirectly by cellular restriction factors and includes interferon γ inducible protein 16 (IFI16) (Cristea et al., 2010; Dell'Oste et al., 2014; Li et al., 2012), lysine demethylases (KDMs) (Lee et al., 2015) and promyelocytic leukemia (PML) nuclear bodies (Landolfo et al., 2016). Viral proteins also influence MIEP regulation. UL82 (pp71) is delivered in virions and inhibits PML suppression of the MIEP by degrading daxx (Bresnahan and Shenk, 2000; Hofmann et al., 2002; Ishov et al., 2002) and promotes viral replication (Figure 2). The activating and inhibitory functions of these factors on the MIEP play a fundamental role in regulating the switch from latency to lytic infection and *vice versa* and are almost certainly cell type and context dependent (Goodrum, 2016; Elder and Sinclair, 2019; Forte et al., 2020).

Principal IE genes are transcribed by host RNA polymerase II (pol II) from a single transcriptional start site upstream of the *UL123* and *UL122* genes, and alternatively spliced to yield the IE1 and IE2 families of products respectively, with unique polyadenylation sites (Stenberg et al., 1985; Awasthi et al., 2004; Arend et al., 2016). The IE1 family consists of the abundant 72 kda IE1 (IE1-72) and possibly IE1-19 and IE1-17 products, however the latter two have not been functionally characterised (Awasthi et al., 2004; Paulus and Nevels, 2009). The IE2 family consists of IE2-86, as well as IE2-60 and IE2-40 which are expressed from internal promoters and accumulate to peak levels between 48 and 72 HPI (Stenberg et al., 1989; Puchtler and Stamminger, 1991; Plachter et al., 1993; White et al., 2007; Weekes et al., 2014; Parida et al., 2019; Li M. et al., 2020). In general, IE1-72 and IE2-86, referred to as IE1 and IE2 from here, are multifunctional proteins that transactivate DE viral genes, remodel chromatin, disrupt interferon signalling, and inhibit apoptosis to create a conducive cellular environment for viral replication. IE1 has been shown to be dispensable for replication at high multiplicity of infection (MOI), but essential at low MOI with a failure to accumulate DE transcripts in the mutants, potentially due to autoregulation of the MIEP by IE1 and compensated for by transactivating virion tegument proteins at high MOI (Mocarski et al., 1996; Greaves and Mocarski, 1998; Gawn and Greaves, 2002). IE2 was shown to be essential for accumulation of DE gene products (Marchini et al., 2001; Heider et al., 2002) but recently has been shown to also have important transactivating functions late in infection when peak expression is observed (Li M. et al., 2020). Interestingly, IE2 also negatively regulates expression from the MIEP during late infection through a *cis*-repression sequence (*crs*) within the core promoter (Cherrington et al., 1991; Lang and Stamminger, 1993; Macias and Stinski, 1993), and likely acts as a feedback mechanism to tune the transcriptional program over the course of infection. This is further supported by observations that IE2 both activates and represses transcription from multiple viral loci (Li M. et al., 2020; Ball et al., 2022). The transcriptional regulation mechanisms of IE1 and IE2 are not clear cut, but also include a chromatin dependent contribution (Nevels et al., 2004; Paulus et al., 2010; Nevels et al., 2011; Knipe et al., 2013; Zalckvar et al., 2013). IE1 and IE2 both exert effects on innate immune signalling, with IE1 modulating interferon stimulated gene (ISG) expression primarily through signal transducer and activator of transcription (STAT) dependent mechanisms (Paulus et al., 2006; Huh et al., 2008; Krauss et al., 2009; Knoblach et al., 2011; Reitsma et al., 2013; Harwardt et al., 2016) as well as disrupting PML nuclear bodies (Lee et al., 2004; Scherer et al., 2014; Scherer and Stamminger, 2016), while IE2 broadly blocks cytokine production through STING and NF-kB (Taylor and Bresnahan, 2005; Taylor and Bresnahan, 2006a; Taylor and Bresnahan, 2006b; Kim et al., 2017; Botto et al., 2019). DNA microarray analysis of IE2 expressing cells showed induction of E2 transcription factor (E2F) regulated genes and was postulated to drive the cell cycle from G0/G1 to G1/S (Song and Stinski, 2002), however, this may be indirect as negligible transcription or promoter binding of cellular genes has been observed for IE2 using PRO-seq and ChIP-seq methods (Spector D. H. 2015; Li M. et al., 2020; Ball et al., 2022). Finally, both IE1 and IE2 contribute to apoptosis inhibition (Zhu et al., 1995; Tanaka et al., 1999; Yu and Alwine, 2002; Hsu et al., 2004), and together with the aforementioned functions, establish a conducive cellular environment for HCMV replication (Figure 2).

## Stage 3: Establishment of the nuclear replication compartment

Herpesvirus infections generate an intranuclear structure termed the viral replication compartment (RC) for viral DNA replication. Pre-RCs are visible as multiple distinct puncta from 6 h post infection (HPI) in cell culture, and can be visualised by the UL112/UL113 proteins (Wright et al., 1988; Penfold and Mocarski, 1997; Schommartz et al., 2017). Between 12 and 24 HPI UL44, UL57 and IE2 are visible at RCs, immediately adjacent to PML bodies (Penfold and Mocarski, 1997; Ahn et al., 1999). Between 24 and 96 HPI, viral RCs expand and coalesce into larger structures that usually form a single inclusion that occupies most of the nucleus, although a minority of cells maintain two separate structures on either side of the nucleus (unpublished observations) (Strang, 2015). After RC enlargement, UL44 and host nucleolin associate with the RC periphery (Strang et al., 2010; Strang et al., 2012a). UL84 also associates with the RC periphery and is dependent on nucleolin for correct localisation (Bender et al., 2014), with the n-terminal domain the primary determinant for UL44 interaction (Strang Blair et al., 2012). Nucleolin is also required for correct RC architecture and UL44 localisation, but not for viral DNA synthesis, consistent with a scaffolding function at the RC periphery (Strang et al., 2012b).

**FIGURE 3:**
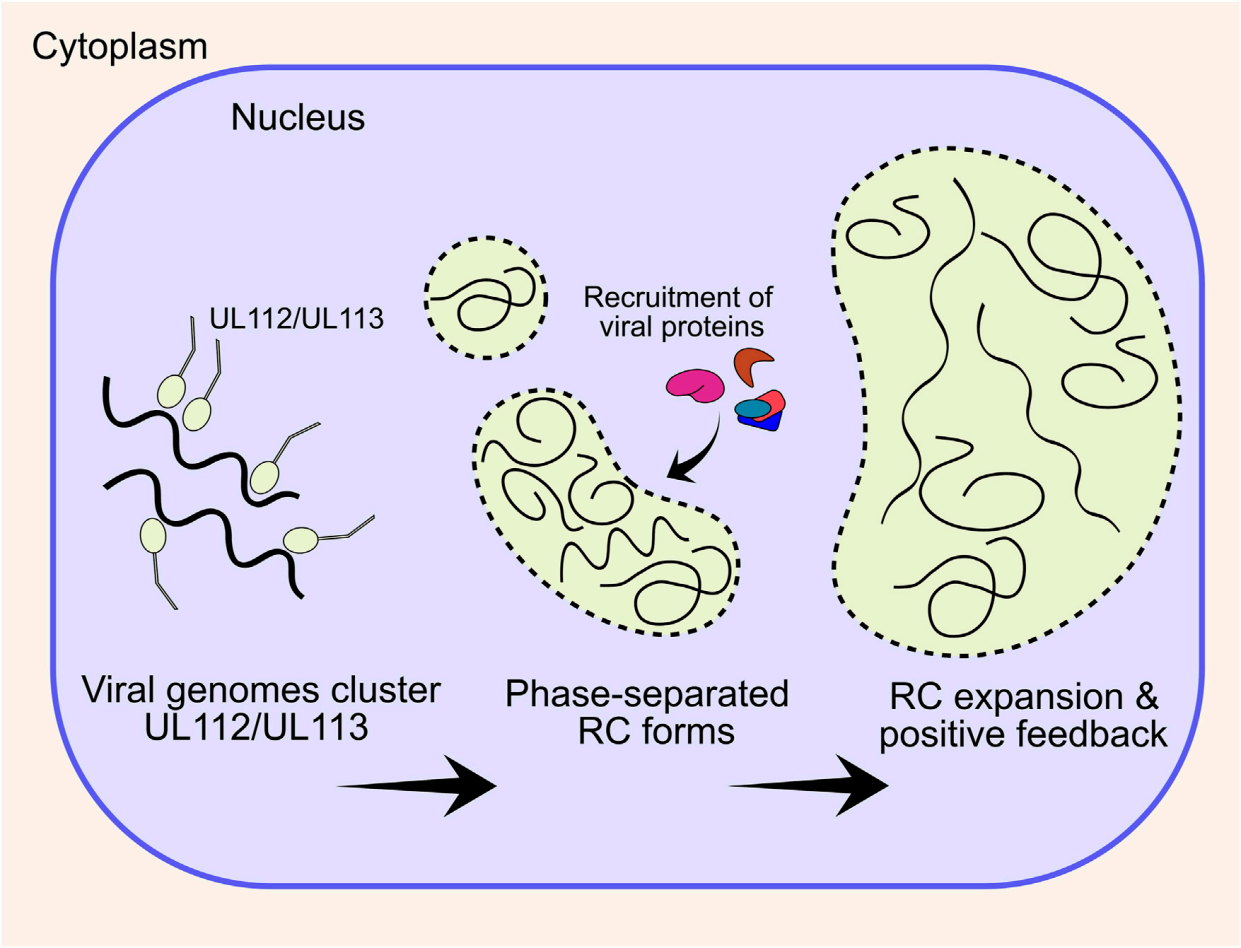
HCMV forms an intranuclear replication compartment for viral DNA replication and transcription. The UL112/UL113 proteins coalesce around viral genomes and together induce a phase separation. Nuclear viral proteins, including DNA replication machinery preferentially localises to the phase separated compartment, enhancing DNA replication and creating positive feedback. Over the course of infection, multiple small pre-RCs expand and coalesce into a single large structure that occupies most of the nuclear volume. RC, replication compartment.

UL112/UL113 is key to RC formation, and co-expression with the 6 core replication fork proteins (discussed in Stage 4) by transient transfection in Vero cells resulted in RC-like staining patterns, whilst no RCs form without UL112/UL113 (Ahn et al., 1999). Further, UL112/UL113 is also needed for correct UL44 localisation and subsequent viral DNA replication (Kim and Ahn, 2010), with the n-terminal domain shown to be essential for this (Kim et al., 2015). UL112/UL113 encodes 4 isoforms, with only p43 and p84 essential for viral replication and UL44 localisation (Schommartz et al., 2017). More recently, it has emerged that HSV-1 and HCMV RCs are molecular condensates that form through a process of liquid-liquid phase separation (LLPS), to create a structure with physical properties that selectively concentrate viral proteins essential for DNA replication, repair and transcription [reviewed (Caragliano et al., 2022a)] (Figure 3). For HCMV, the intrinsically disordered region (IDR) of UL112/UL113 induces LLPS around viral genomes and subsequently recruits essential viral proteins including UL44 (Caragliano et al., 2022b). Over the course of infection, newly replicated viral DNA accumulates within RCs as they expand, together with UL57 (Strang et al., 2012a; Caragliano et al., 2022b). UL112/UL113 also becomes less mobile within RCs over time as RCs display irregular morphologies distinct from their spherical precursors, which is abrogated by DNA synthesis inhibitors (Caragliano et al., 2022b) (Figure 3). This is strong evidence that DNA itself contributes to the physical properties of the RC. However, this is not the sole determinant of RC morphology at late times after DNA replication, as deletion of the RC resident protein UL34 had no impact on the levels of viral DNA, but altered the size, morphology, and electron density of RCs (Turner et al., 2022a). Further, fluorescent labelling of viral genomes showed colocalization with maturing capsids at the RC periphery, and potentially indicates that genome replication (Stage 4) and packaging (Stage 7) is coupled, at least at late times (Mariamé et al., 2018). Interestingly, maturing capsids also mis-localise when RC residents UL34 and UL84 are deleted (Strang Blair et al., 2012; Turner et al., 2022a), further indicating that the RC periphery is involved in capsid maturation and genome packaging, although precise mechanisms currently remain elusive.

## Stage 4: Genome replication

**FIGURE 4:**
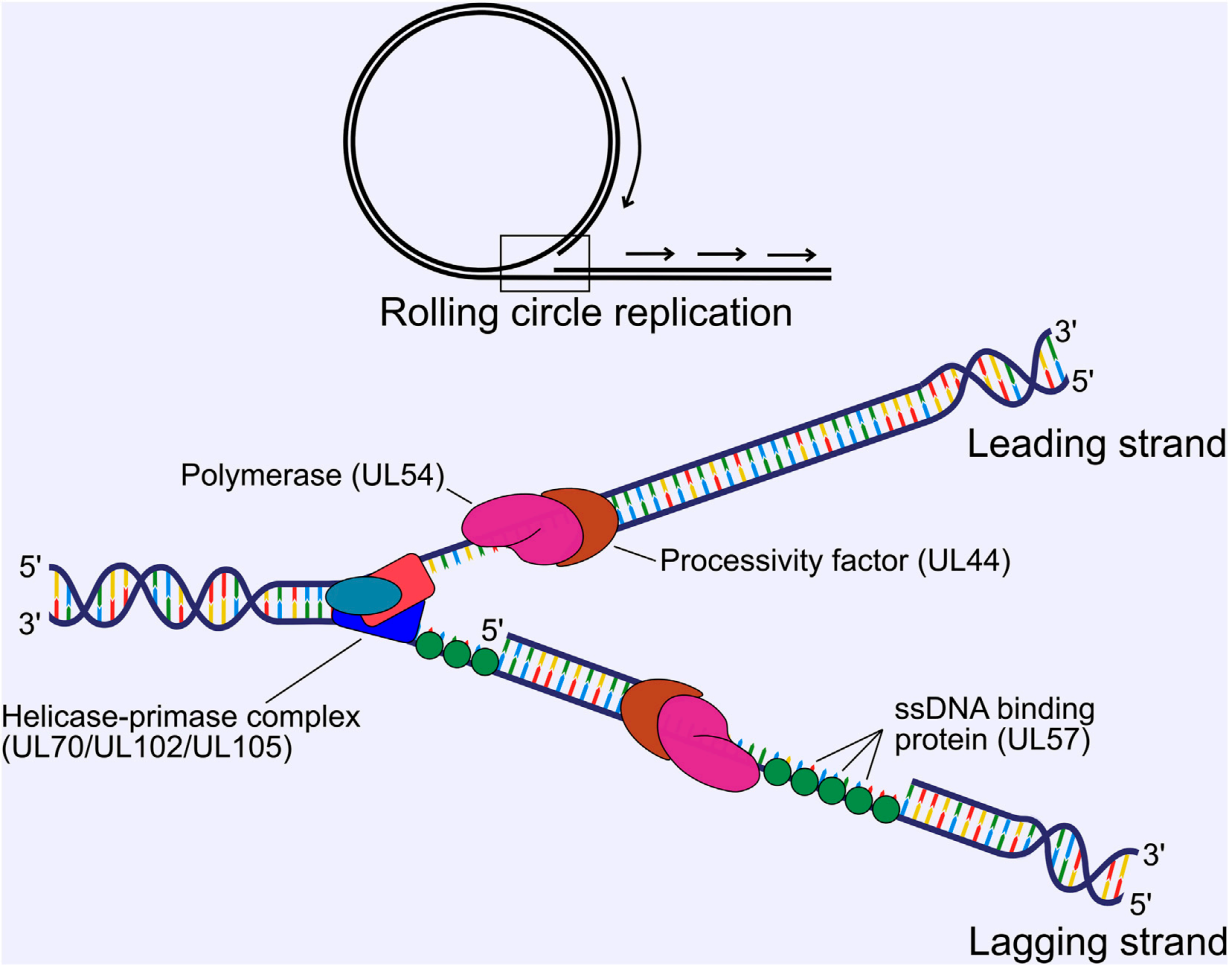
Viral DNA replication proceeds by a rolling circle mechanism to produce linear concatemers. The tripartite helicase-primase complex composed of UL70, UL102 and UL105 separates the strands to form a replication fork, while the DNA polymerase (UL54) and processivity factor (UL44) synthesise the daughter strands by leading and lagging strand synthesis. The ssDNA binding protein UL57 forms filaments on ssDNA, which stimulates polymerase and primase activity. ssDNA, single stranded DNA.

HCMV genome replication proceeds from the origin of lytic replication (*oriLyt*) situated immediately upstream of *UL57* between 91,000 and 94,000 bp in the genome (Masse et al., 1992; Borst and Messerle, 2005), and is thought to occur by a rolling circle mechanism that produces lagging strand loops termed the “trombone” mechanism, based on HSV-1 (Bermek et al., 2015). Rolling circle replication produces linear, concatenated viral genomes. Genome replication is performed by 6 virally encoded replication fork proteins. For HCMV, these were inferred from homology of the HSV-1 polymerase complex (Weller and Coen, 2012; Packard and Dembowski, 2021) and were confirmed functionally in HCMV by Pari et al. using a transient complementation-based assay (Pari and Anders, 1993; Pari et al., 1993). These consist of the DNA polymerase catalytic subunit (UL54), the polymerase processivity factor (UL44), single-stranded DNA-binding protein (UL57), and the tripartite helicase-primase complex composed of UL70, UL102 and UL105 (McMahon and Anders, 2002). UL54 and UL44 interact (Ertl and Powell, 1992; Nobre et al., 2019) and together efficiently catalyse DNA synthesis (Weiland et al., 1994) (Figure 4). Structural characterisation of HCMV UL44 showed it had strong homology to HSV-1 UL42 (UL44 homologue), and may function analogously to mammalian proliferating cell nuclear antigen (PCNA) (Appleton et al., 2004), with the UL54-UL44 interaction mapped to the c-terminus of UL54 and the “connector loop” of UL44 (Loregian et al., 2003; Loregian et al., 2004a; Loregian et al., 2004b; Appleton et al., 2006). Few HCMV specific studies have been performed to elucidate the mechanistic contribution of UL57, UL70, UL102 and UL105 to DNA replication, and their function has been largely assigned based on HSV-1 [reviewed (Mercorelli et al., 2008; Packard and Dembowski, 2021)]. In this scheme, the helicase unwinds dsDNA to form a replication fork and the primase synthesises short RNA primers to initiate lagging strand synthesis. The polymerase and processivity factor synthesise daughter strands by leading and lagging strand synthesis. UL57 forms filaments on ssDNA to stimulate polymerase and helicase-primase function and may possess additional strand annealing activity involved in DNA recombination, based on HSV-1 function (Tolun et al., 2013; Weerasooriya et al., 2019) (Figure 4). No structure has yet been solved for the helicase-primase complex, but some conserved functional motifs have been identified (Woon et al., 2008; Ligat et al., 2018a).

*Trans*-complementation experiments by Pari et al. identified a total of 11 loci as essential for *oriLyt* dependent DNA replication (Pari and Anders, 1993; Pari et al., 1993). In addition to the 6 core replication fork components described above, the IE1/IE2 locus, UL36-38, IRS1/TRS1, UL112/UL113 and UL84 were found to be essential. IE1/IE2, UL36-38 and IRS1/TRS1 (see Stage 2), as well as UL112/UL113 (see Stage 3) all contribute to transactivate core replication machinery or create a permissive cellular environment at early times, and likely do not play a direct role in DNA replication. Herpesviruses encode origin binding proteins that bind to the *oriLyt* and initiate DNA replication. UL84 is essential for replication in cell culture for lab adapted strains (Dunn et al., 2003; Yu et al., 2003), and extensive experimentation lead to the model that UL84 fulfils this role for HCMV and is dependent on IE2 [reviewed (Pari et al., 2008)]. It was subsequently revealed that HCMV strains TR and TB40E replicate independently of UL84, and this ability was mapped to a single amino acid change within IE2 (H388D), that rescued growth of UL84 dependent strains with UL84 deletion (Spector D. J. 2015). Further, TB40E UL84 deletion mutants can replicate plasmids containing *oriLyt* sequence from a UL84 dependent strain (Manska and Rossetto, 2021). The exact determinants of HCMV DNA replication initiation are yet to be resolved but may depend on IE2 transcriptional activation and repression from the *oriLyt* promoter region. Herpesvirus DNA replication also requires a DNA-RNA hybrid within the *oriLyt* termed an “R-loop” (Wang et al., 2006; Rennekamp and Lieberman, 2011). For HCMV, this is the G-C rich 5’ region of the long non-coding RNA4.9 which may be required to initiate origin binding of the replication machinery (Tai-Schmiedel et al., 2020). Interestingly, this region strongly binds AD169 UL84, but not TB40E UL84, but no difference was observed for IE2 occupancy between strains, and fails to explain the ability of IE2 (H388D) to initiate DNA replication (Manska and Rossetto, 2021). More work is needed to solve this intricate mechanism of replication initiation. Finally, host proteins also influence HSV-1 DNA replication and include DNA damage and repair proteins, topoisomerases, transcription factors and chromatin remodelling proteins [reviewed (Packard and Dembowski, 2021)]. Recent work on HCMV has identified a similar repertoire of host proteins compared to HSV-1, and await functional characterisation (Manska and Rossetto, 2022).

## Stage 5: Late gene expression

**FIGURE 5:**
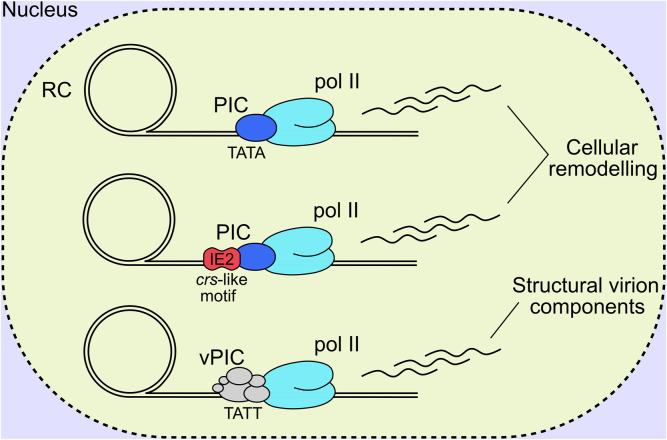
HCMV late genes are expressed with comparable kinetic profiles, however, distinct mechanisms of transcriptional initiation exist. The first involves TATA binding by host TBP, recruitment of a canonical PIC and RNA pol II, and transcription initiation akin to the alpha-herpesviruses. The second involves IE2-based regulation of transcriptional initiation, by binding to a *crs*-like motif and recruiting the host PIC and pol II for gene transcription. It must be noted that IE2 is a multifunctional protein that likely influences viral gene activation, repression, and as an elongation barrier depending on the promoter sequence, bound transcription factors and local chromatin environment. The third mechanism is unique to the beta- and gamma-herpesviruses that encode a 6-member vPIC that binds to unconventional TATT promoter sequences and recruits host pol II for transcript elongation. RC, replication compartment; IE2, immediate early 2; TBP, TATA binding protein; PIC, pre-initiation complex; vPIC, viral pre-initiation complex; pol II, host RNA polymerase II complex.

Herpesviral late genes are transcribed by RNA pol II, and predominantly encode structural virion components, with their classification defined by robust accumulation at late times following viral DNA replication. Canonically, there are two distinct late classes for all herpesviruses termed leaky late (LL) and true late (TL), or gamma 1 and gamma 2, respectively (Anders et al., 2007). LL products are generally understood to be transcribed from a canonical TATA box promoter motif, and expressed independently of DNA replication; however, their levels are substantially lower in cells treated with viral DNA polymerase inhibitors, and likely due to low template numbers in this condition (Figure 5). In contrast, TL products are canonically defined as being entirely dependent on DNA replication (Gruffat et al., 2016). This suggests that changes occur to viral DNA following replication, with the removal of repressive chromatin and sequestration of replicated genomes in the interior of viral RCs the current favoured hypotheses (Gruffat et al., 2016). Despite all herpesviruses having LL and TL expression classes with superficially similar characteristics, there are mechanistic differences in the transcriptional initiation of late gene products between the alpha, and the beta and gamma subfamilies. Namely, alphaherpesviruses initiate TL gene expression by tuning host pre-initiation complex (PIC) recruitment to late promoters, likely through a combination of the TATA box, initiator element and early viral transactivators (Mavromara-Nazos and Roizman, 1989; Kim et al., 2002; Dremel and DeLuca, 2019). In contrast, the beta and gamma subfamilies have a 6-member virally encoded PIC (vPIC) which recognises a non-canonical TATT motif in core promoters which subsequently recruits pol II for transcriptional initiation (Tang et al., 2004; Isomura et al., 2008; Gruffat et al., 2012; Aubry et al., 2014; Gruffat et al., 2016; Li M. et al., 2021) (Figure 5). The 6 sub-units composing the HCMV vPIC are UL49, UL79, UL87, UL91, UL92 and UL95 (Isomura et al., 2011; Perng et al., 2011; Omoto and Mocarski, 2013; Omoto and Mocarski, 2014; Turner et al., 2022b) all of which are essential for replication in cell culture (Dunn et al., 2003; Yu et al., 2003). UL87 is central to the complex and is thought to bind DNA directly based on a predicted TATA binding protein (TBP) fold (Wyrwicz and Rychlewski, 2007) and gammaherpes analogues (Wong et al., 2007; Gruffat et al., 2012; Davis et al., 2015).

Recently, PRO-seq was performed at late time of infection following degradation of IE2 isoforms and vPIC subunits to profile different transcription initiation mechanisms across the viral genome (Li M. et al., 2020; Li M. et al., 2021). Degradation of IE2 isoforms IE-86, IE-60 and IE-40 blocked transcription initiation for 42 transcripts and repressed 7, with both LL and TL expression kinetics (Li M. et al., 2020). The majority of IE2 activated transcripts did not possess a TATW (W = A or T) motif with 5 TATA and 5 TATT box containing promoters sensitive to IE2 activation (Li M. et al., 2020). The same methodology, when applied to degradation of vPIC subunits UL79 and UL87, showed a substantial reduction in viral transcription initiation globally, and included promoters with both LL and TL expression kinetics (Li M. et al., 2021). Complementing these results, RNA-seq profiling revealed a consistent diminution of transcripts encoding structural virion and egress associated products in vPIC mutant infections (Turner et al., 2022b). Analysis of vPIC sensitive promoters revealed the TATTW (W = A or T) motif to be enriched, and is concordant with gammaherpesvirus results (Tang et al., 2004; Wyrwicz and Rychlewski, 2007; Gruffat et al., 2012; Aubry et al., 2014). However, multiple additional non-canonical motifs were also present (Li M. et al., 2021). vPIC regulated transcripts showed a positive correlation with viral polymerase inhibitor phosphonoformic acid (PFA) sensitivity (Li M. et al., 2021), while IE2 regulated transcripts displayed a wide range in PFA sensitivity (Li M. et al., 2020), substantiating the notion that the HCMV vPIC broadly shapes the late transcriptional program with a smaller contribution from IE2. Additionally, IE2 and vPIC regulation appears to be specific to viral genomes, with neither substantially altering host transcription (Li M. et al., 2020; Li M. et al., 2021). This could be due to unrecognised cis-regulatory elements in the viral genome, or more likely, sequestration of viral DNA replication, transcription and altered chromatin status within nuclear RCs (Stage 3) where vPIC subunits localise (Isomura et al., 2011; Perng et al., 2011; Omoto and Mocarski, 2013; Omoto and Mocarski, 2014). Analysis of IE2 occupancy on the genome suggested IE2 functions as a transcriptional repressor, activator and elongation barrier depending on the promoter sequence, bound transcription factors and local chromatin environment, likely modulated by protein-protein interactions as well as DNA sequence directly depending on the context (Ball et al., 2022) (Figure 5). Recent work using DNA fragmentation factor (DFF) before immunoprecipitation (DFF-ChIP), which analyses the run-on products of protected fragments after DNA digestion and immunoprecipitation of selected proteins, has revealed unprecedented insights into chromatin status and mechanisms of transcription initiation of the viral genome (Spector et al., 2022). It was shown that 85% of pol II was not associated with +1 nucleosomes (ie. stalled transcription) on HCMV promoters, compared to only 18% on host promoters, and that viral DNA has 100 times less H3K4me3 modified nucleosomes compared to host DNA. Taken together, HCMV genes are predominantly transcribed from unchromatinised DNA (Parida et al., 2019; Spector et al., 2022), at least at late times, which is concordant with RC sequestration of viral genomes (Caragliano et al., 2022a; Caragliano et al., 2022b). Additionally, comparison of transcription from viral promoters for host TBP PICs and UL87 vPICs over time using PRO-seq, revealed that the vPIC preferences kinetically late and PFA sensitive promoters as previously described (Li M. et al., 2021). However, some promoters were robustly transcribed using only host TBP PICs at late times (Spector et al., 2022). Most striking is that both TBP and UL87 have activity on many viral promoters and likely compete for occupancy, exemplified by UL22A, which reveals a dynamic interplay between separate transcription initiation mechanisms on the HCMV genome (Spector et al., 2022).

Herpesviral gene expression kinetics were traditionally divided into IE, E and L gene products but this view has shifted with investigations utilising “omics” technology. Weekes et al. profiled the host and HCMV proteome over 96 h of lytic replication, with and without PFA treatment, and defined 5 kinetic expression classes (Weekes et al., 2014). RNA-seq profiling over time yielded similar groupings with 5 classes (Stern-Ginossar et al., 2012). Recently, RNA-seq profiling, in combination with PFA and cycloheximide (translation inhibitor), revealed viral transcription does not proceed in a sequential temporal cascade but instead, at most time points, there are transcripts sensitive to different drugs with similar profiles (Rozman et al., 2022). The authors used these data to suggest 7 temporal expression classes (Rozman et al., 2022). Different isoforms also show different mechanisms of regulation at early and late times [reviewed (Hale and Moorman, 2021)]. UL44 is a prominent example as it is expressed early (Leach and Mocarski, 1989), but peak transcript and protein levels are reached late (Weekes et al., 2014; Rozman et al., 2022). UL44 has 3 transcription start sites, the second containing a vPIC dependent TATT motif (Leach and Mocarski, 1989; Isomura et al., 2007; Isomura et al., 2008; Isomura et al., 2011), which likely governs these dynamics. IE2 and UL82 behave similarly with late isoforms transcribed by the vPIC from TATT containing promoter elements (Li M. et al., 2020; Li M. et al., 2021). Reconciling temporal expression profiles with recent insights of late transcriptional regulation by TBP, IE2 and vPIC, and their preferences and competition for varied promoter sequences, revealed a complex and dynamic late transcriptional landscape that was not previously appreciated (Figure 5). It is clear that early/late classification is inadequate to account for the observed complexity, as is a definition based solely on transcriptional initiation mechanism or promoter motif enrichment, given the sequence diversity of each PIC and competition or redundancy between these (Parida et al., 2019; Li M. et al., 2020; Li M. et al., 2021; Ball et al., 2022; Spector et al., 2022). Given the potential promoter competition between TBP and vPIC at some promoters, it is conceivable that substantial compensatory effects may exist depending on the context, such as in mutant virus infections, certain drug treatments or cell type, which may help or hinder interpretation depending on the context. There is now a substantial body of data available relating to late transcription which is ripe for integration and re-analysis, to reveal new insights and answer outstanding questions (Jürges, 2022).

## Stage 6: Biogenesis of the viral assembly compartment

**FIGURE 6:**
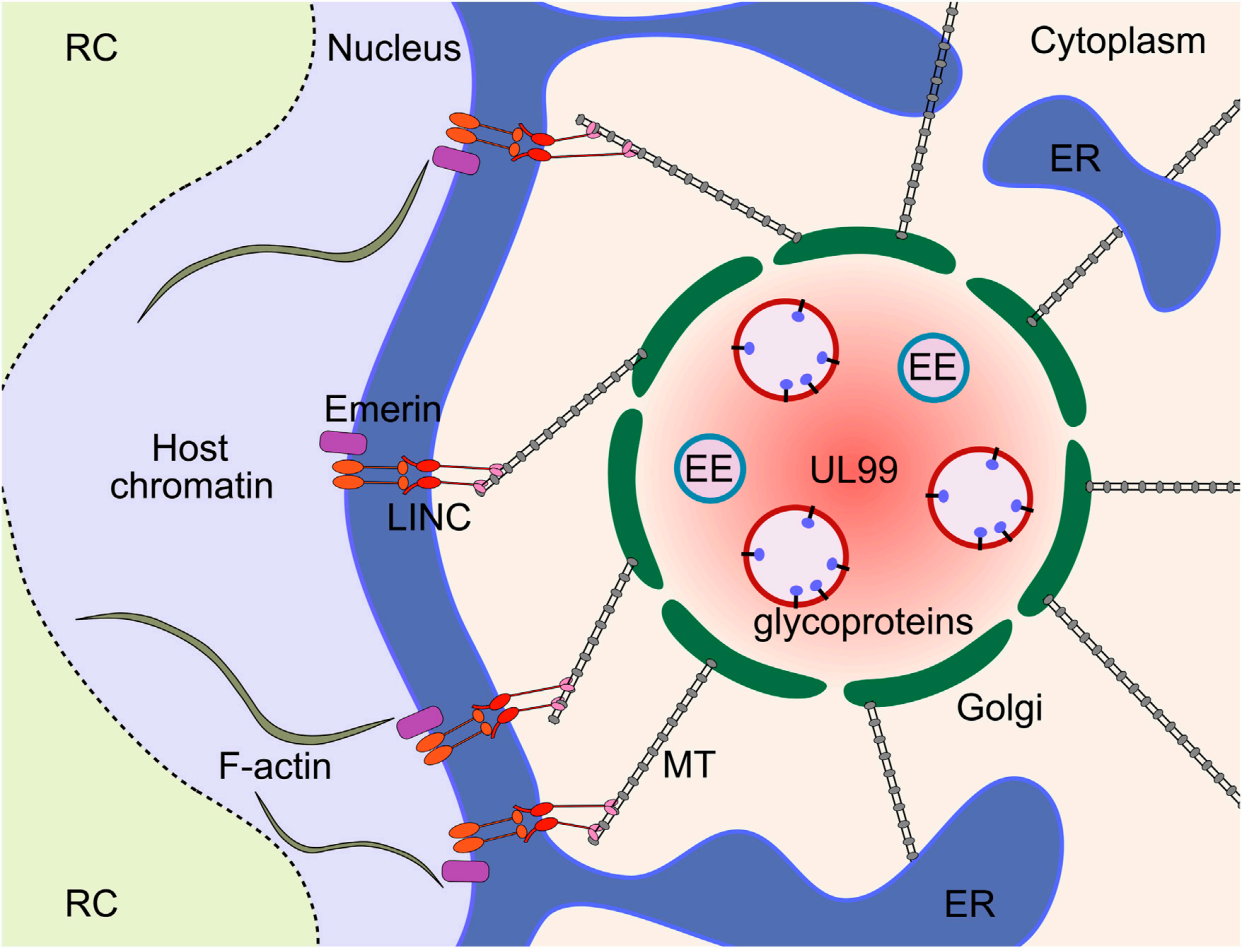
The HCMV vAC is a cytoplasmic virus factory where virion cargo accumulates to enable tegumentation and secondary envelopment. The vAC is characterised by concentric rings of host-derived organelles, with endosome membranes surrounded by a Golgi ring, and the ER loosely associated around the periphery. The structure is adjacent to the enlarged kidney-shaped nucleus and functions as a Golgi-derived MTOC. The cytoskeleton is central to vAC formation and function, and is connected to the nucleus through polarised LINC complexes. LINC complexes provide a bridge between the nucleoskeleton and cytoskeleton that allows the vAC to exert control over nuclear morphology, rotation, and internal organisation. ER, endoplasmic reticulum; EE, early endosome; MT, microtubule; MTOC, microtubule organising centre; LINC, linker of nucleoskeleton and cytoskeleton; vAC, viral assembly compartment; RC, replication compartment.

HCMV infected cells generate a cytoplasmic megastructure known as the viral assembly compartment (vAC), that serves as an essential organisational hub for virion cargo recruitment, tegumentation, secondary envelopment, cytoplasmic trafficking, and release of infectious virions [reviewed (Alwine, 2012; Jean Beltran and Cristea, 2014; Close et al., 2018)]. The vAC was first described by Sanchez et al., and was postulated to be the site of final virion assembly (Sanchez et al., 2000a; Sanchez et al., 2000b). The major viral markers of the vAC include the tegument protein UL99 and envelope glycoprotein gB, however, most virion cargo proteins including gH, UL32 and UL83 localise to the vAC at late times during infection (Sanchez et al., 2000a; Sanchez et al., 2000b). Utilising a panel of organelle specific markers, Das and others further characterised the host cell features of the vAC, and proposed a vAC model that takes the form of a flattened disc composed of concentric rings of organelles centred on a microtubule organising centre (MTOC), with the whole structure adjacent to an enlarged kidney-shaped nucleus (Das et al., 2007; Das and Pellett, 2007; Das and Pellett, 2011; Das et al., 2014). Since HCMV inactivates centrosomes (Hertel and Mocarski, 2004), it was recently shown that the vAC functions as a Golgi derived MTOC (Procter et al., 2018). Endosomal vesicles are clustered within a ring of Golgi membranes with the endoplasmic reticulum (ER) loosely associated around the periphery (Das et al., 2007; Das and Pellett, 2007; Das and Pellett, 2011; Das et al., 2014) (Figure 6). Recent work has suggested that there are multiple populations of heterogeneous vesicles clustering within the vAC, potentially containing distinct microdomains (Zeltzer et al., 2018), consistent with the extensive re-modelling of host organelles throughout infection (Jean Beltran et al., 2016). Organelle remodelling and vAC formation is also observed in murine CMV (MCMV) infected cells, with all the same features as HCMV (Lučin et al., 2020; Marcelić et al., 2021; Pavišić et al., 2021; Štimac et al., 2021), showing important conservation. Observations of HCMV infection by live cell imaging has provided unprecedented insights into the dynamic nature of this structure (Procter et al., 2018). The authors observed vACs splitting and merging throughout infection, while the nucleus was in constant motion around the vAC, often re-orienting and rotating through 360° (Procter et al., 2018).

The vAC is a highly complex and dynamic structure however, the host and viral dependencies remain largely uncharacterised, and only a handful identified to date. Initial efforts utilised siRNA to knockdown (KD) 26 selected viral proteins of interest, from early and late kinetic classes (Das et al., 2014). The screen identified and validated UL48 and UL103 to be prerequisite for typical endosome accumulation and Golgi ring formation, but no detailed mechanistic information was revealed. UL103 deletion was shown to influence vAC morphology, but direct contribution to vAC organisation separate from its role in egress was not assessed (Ahlqvist and Mocarski, 2011). Likewise, deletion of UL71 (Womack and Shenk, 2010), UL94 (Phillips et al., 2012) and UL97 (Azzeh et al., 2006) show disruption of select vAC markers but this may be secondary to defects in envelopment (Womack and Shenk, 2010; Goldberg et al., 2011; Schauflinger et al., 2011; Phillips and Bresnahan, 2012; Dietz et al., 2018; Read et al., 2019), as well as trafficking of UL99 in the case of UL94 (Phillips et al., 2012) (see Stage 9). Deletion of the envelope glycoprotein UL132 was previously demonstrated to reduce virus titres by 100-fold (Spaderna et al., 2005) and has recently been shown to be essential for vAC formation (Wu et al., 2020). The virions released from UL132 deletion infections had high particle/PFU ratios and altered virion cargo composition, namely reduced levels of envelope glycoproteins gH and gB, and outer tegument proteins UL71 and UL99 (Wu et al., 2020). Virion production, vAC formation, particle/PFU ratio and virion composition were rescued by expression of the cytoplasmic domain in trans which demonstrates the entry defects are not due to UL132 in the virion envelope, as rescued virions lack envelope UL132 (Wu et al., 2020). HCMV also encodes multiple miRNAs that target components of the endocytic system (Hook et al., 2014), and deletion of these during infection abrogated vAC formation, and increased particle to PFU ratios analogous to UL132 deletion (Wu et al., 2020).

Several host proteins have been identified to play important roles during vAC biogenesis. Examples include the Golgi residents syntaxin 5 (STX5) (Cruz et al., 2017) and Golgi reassembly stacking protein 65 kD (GRASP65) (Rebmann et al., 2016), ER chaperone BiP (Buchkovich et al., 2008; Buchkovich et al., 2009), and nuclear WDR5 that translocates to the vAC during HCMV infection (Yang et al., 2021; Yang et al., 2022). Proper functioning of the host endocytic system is required for vAC formation (Archer et al., 2017), including dynamin (Hasan et al., 2018; Štimac et al., 2021). Endocytic involvement is further supported by the localisation and requirement for ADP ribosylation factor 1 (ARF1), ARF3, ARF4 and ARF6 GTPases for establishment of pre-ACs in MCMV infection (Donaldson and Jackson, 2011; Pavišić et al., 2021). The ras-related in brain 11 (RAB11) family interacting protein 4 (FIP4) interacts with the gM cytoplasmic tail and all three are required for correct vAC organisation and efficient virus production (Krzyzaniak et al., 2009). Bicaudal D1-dependent localisation of UL32 at late times, but not UL99, further illustrates the specificity and general importance of trafficking within the vAC (Indran et al., 2010). Trafficking within the vAC is complex with a major host cell contribution and exact mechanisms for each of these has not yet been determined.

Given that the vAC functions as a Golgi derived MTOC (Procter et al., 2018), it is not surprising that the cytoskeleton is also a major determinant of vAC architecture. Treatment of infected cells with the microtubule (MT) depolymerising drug nocodazole disrupts the vAC (Sanchez et al., 2000a), which is reversible following removal (Indran et al., 2010). Mechanistically, the MT associated end binding protein 1 (EB1) and EB3 are both required for efficient HCMV replication, but function through distinct mechanisms (Procter et al., 2018). EB1 depletion caused mislocalisation of the MT plus end tracking protein CLIP170 along the MT lattice, while EB3 depletion abrogated formation of acetylated MTs. Additionally, HCMV requires transforming acidic coiled-coil containing protein 3 (TACC3), an EB-independent plus end tracking protein which recruits the MT polymerase chTOG for correct vAC architecture and efficient replication (Furey et al., 2021). The cytoplasmic vAC has previously been linked to effects on nuclear morphology, with contributions from BiP (Buchkovich et al., 2010), and strengthened by the knowledge that acetylated MTs are required for nuclear rotation that is blocked in EB3 knock-down conditions (Procter et al., 2018). These observations were extended by Procter et al. (Procter et al., 2020) that established that HCMV polarises the nuclear membrane linker of nucleoskeleton and cytoskeleton (LINC) complexes towards the vAC, *via* contact with acetylated MTs through dynein to regulate nuclear morphology and rotation (Procter et al., 2020). Additionally, intranuclear organisation is controlled by the cytoskeleton through the LINC. Polarised LINC complexes alter the localisation of F-actin which segregates host heterochromatin towards the vAC, while viral DNA is localised away from the vAC in RCs (Stage 3) (Figure 6). Taken together, these finding establish that the vAC exerts control over nuclear morphology, rotation, and organisation, and that the nuclear RC and vAC are inherently linked (Procter et al., 2020). Together, this work definitively shows that the vAC acts as a hub to coordinate efficient virion assembly and egress by remodelling host membranes and the secretory system. Elucidating exact mechanisms of vAC function is complicated with many important proteins likely performing dual roles.

## Stage 7: Capsid assembly and genome packaging

The pseudo-icosahedral nucleocapsid displays T16 symmetry and is composed of 150 major capsid protein (MCP) hexons, with 11 MCP pentons making up each vertex of the icosahedron (Liu and Zhou, 2007; Gibson, 2008). The 12th vertex is the portal which is composed of 12 copies of the portal protein (UL104) arranged in two hexameric rings, creating a channel for the genome to enter and exit the nucleocapsid (Dittmer et al., 2005; Li Z. et al., 2021). A single copy of triplex capsid protein 1 (TRX1, UL46) and 2 copies of TRX2 (UL85) form heterotrimers which fasten the MCP pentamers and hexamers together at their floor (Yu et al., 2017). A single smallest capsid protein (SCP) copy sits atop each MCP copy which provides the interaction surface for the betaherpes specific UL32/pp150 tegument protein to buttress the pentamers and hexamers to the triplex floor, and is postulated to provide extra support to withstand the greater internal pressure from the large HCMV genome that is packaged within (Yu et al., 2017). Recently, the structure of capsid vertex specific components (CVSC) were solved, revealing heteropentamers composed of 2 copies of UL77, two of UL48 and a single UL93 molecule (Li Z. et al., 2021). CVSCs variably occupy the peripenton registers, displacing 3 copies of UL32 and sit atop the triplexes at these locations. The periportal registers are saturated with 5 CVSCs and replace 4 copies of UL32 at each (Li Z. et al., 2021). Interestingly, only 13% of peripenton registers are occupied by CVSC in HCMV (Li Z. et al., 2021), compared with 20% for Epstein-Barr virus (EBV) (Li Z. et al., 2020), 38% for Kaposi’s-sarcoma associated herpesvirus (KSHV) (Gong et al., 2019), and 100% for HSV-1 (Liu et al., 2019), which is inversely correlated with genome size and has previously been suggested to increase capsid pressure to control pressure balance for optimal genome ejection through the NPC (Li Z. et al., 2020). A portal cap sits atop the portal channel to secure the packaged genome and is formed by 5 dimers of UL77 head domains, likely originating from each of the periportal CVSCs (Liu et al., 2019; Liu et al., 2020; Li Z. et al., 2021).

**FIGURE 7:**
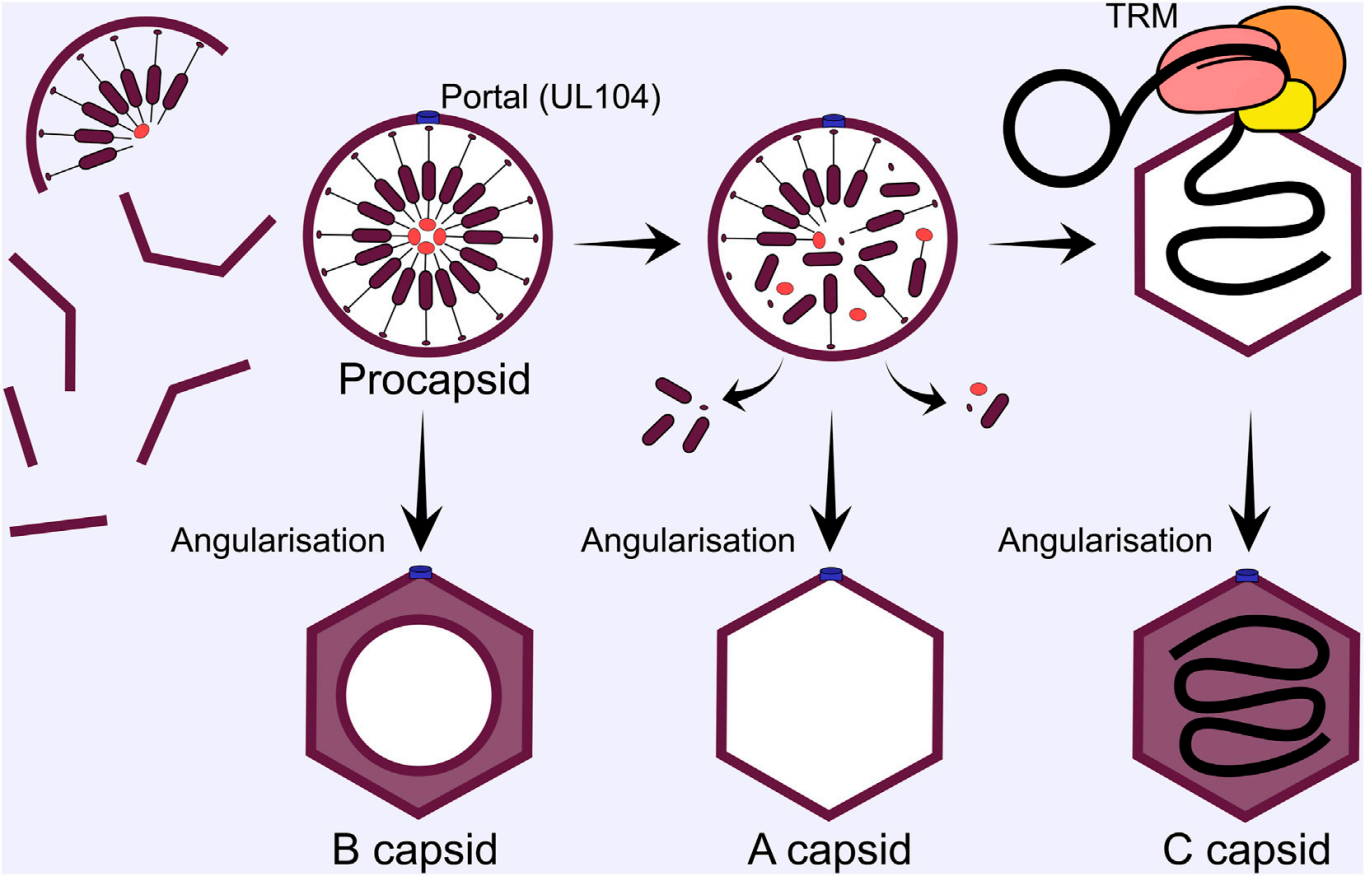
HCMV capsid subunits self-assemble around the scaffold in the nucleus. Once the spherical procapsid is fully assembled, divergence in capsid maturation pathways can occur. If the procapsid angularises to the icosahedral form before the scaffold is removed a B capsid is formed that cannot undergo subsequent maturation. Next, the protease cleaves the scaffold to release it from the interior of the procapsid, and it is ejected through enlarged hexon pores. If angularisation occurs after this step, but before terminase complex engagement, an empty A capsid is formed. Mature C capsids are formed when the terminase complex successfully engages the capsid and delivers a genome. The terminase complex provides energy for genome translocation through ATP hydrolysis, and cleaves unit length genomes from the newly replicated concatemers by endonuclease activity. TRM, tripartite terminase complex.

Capsid formation and maturation occurs in cell nuclei around the periphery of RCs (Mariamé et al., 2018), with important scaffolding and protease proteins involved. The UL80 ORF encodes a fusion protein of the n-terminal protease (UL80a, aa 1-256), linker, and c-terminal scaffold precursor (UL80.5, aa 336-708) (Varnum et al., 2004). There is an alternative UL80.5 transcript that only encodes the scaffold, likely to achieve correct stoichiometry between protease and scaffold (Welch et al., 1991; Loveland et al., 2007; Li M. et al., 2021). The scaffold protein contains two nuclear localisation sequences required for efficient nuclear import of MCP and other capsid proteins (Nguyen Nang et al., 2008). In the nucleus, capsid components assemble around the scaffold to produce a spherical pro-capsid (Figure 7). The scaffold catalyses this by self-interactions *via* the n-terminus, while the c-terminus interacts with MCP (Beaudet-Miller et al., 1996; Wood et al., 1997). These interactions likely underpin HCMV pro-capsid assembly and are analogous to HSV-1 (Gibson, 2008; Brown and Newcomb, 2011). After pro-capsid formation, the protease domain of the fusion protein self-cleaves, releasing the n-terminus, which in turn cleaves the MCP interacting c-terminus to release it from the interior of the capsid shell [reviewed (Gibson, 2008)] (Figure 7). For HCMV, capsid angularisation has been observed when the protease is inhibited, suggesting that the protease is non-essential for this step. However, DNA filled capsids were not observed, confirming the essential function of the protease for further capsid maturation (Yu et al., 2005). HSV-1 procapsids spontaneously assembly *in vitro* and in insect cells when all capsid proteins are expressed (Newcomb et al., 1999). Interestingly, a greater proportion of procapsids angularised in cell extracts, suggesting cellular determinants or co-factors regulate this process (Newcomb et al., 1994; Newcomb et al., 1996; Newcomb et al., 1999). These experiments have not been performed for HCMV and all the viral determinants of pro-capsid formation await confirmation (Gibson, 2008).

Structural comparisons between HSV-1 procapsids and angularised capsids revealed different capsid floor arrangements, with larger pores in the procapsid hexons (Heymann et al., 2003; Brown and Newcomb, 2011). The authors suggested that cleaved scaffold may exit the capsid through these during angularisation (Brown and Newcomb, 2011). During all herpesvirus infections, 3 capsid forms are observed in infected cell nuclei: A-capsids are empty and lack scaffold or DNA, B-capsids contain an inner shell of cleaved scaffold, and C-capsids that contain packaged viral genomes and mature into infectious virions (Tandon et al., 2015). The exact maturation process has not been established experimentally, but a working model has been proposed (Heymann et al., 2003; Baines, 2011). If the tripartite terminase is engaged during angularisation, a genome is packaged to produce a C-capsid. If angularisation occurs sealing the scaffold inside a B-capsid is formed. While if the capsid is sealed after scaffold exit but without a genome packaged, A-capsids result (Heymann et al., 2003; Baines, 2011) (Figure 7).

HCMV genome replication (Stage 4) produces concatemeric genomes that need to be processed. The tripartite terminase complex historically composed of UL51, UL56 and UL89 cleaves and packages unit length genomes into maturing capsids [reviewed (Ligat et al., 2018b)], in a process analogous to that of tailed bacteriophages (Rao and Feiss, 2015) (Figure 7). UL56 is the large subunit and interacts with the capsid portal (Dittmer et al., 2005), specifically binds the *pac1* and *pac2* DNA motifs in the a’ region of the viral genome (Bogner et al., 1998) and is thought to transduce the energy for translocation of the genome given homology to human topoisomerase I (Visalli et al., 2015) and ATPase activity (Hwang and Bogner, 2002; Scholz et al., 2003; Wang and McVoy, 2008). UL89 is the small subunit which performs DNA cleavage (Scheffczik et al., 2002; Couvreux et al., 2010; Nadal et al., 2010; Theiß et al., 2019) and likely interacts with the c-terminal domain of UL56 (Ligat et al., 2017). The third subunit, UL51, was shown to be essential for genome cleavage and packaging, and interacts with UL56 and UL89 (Borst et al., 2013). Moreover, sequestration of all three subunits in the complex protects others from proteosomal turnover (Neuber et al., 2017). UL56 contains a nuclear localisation sequence (NLS) (Giesen et al., 2000) and can localise to the nucleus when expressed exogenously, whilst UL51 and UL89 require UL56 interaction for correct nuclear localisation (Wang et al., 2012; Neuber et al., 2017). UL52 is essential for genome cleavage-packaging and has distinct intranuclear localisation compared to the terminase sub-units (Borst et al., 2008). Conserved domains were identified by polymorphism analysis with putative CXXC-like and zinc finger motif residues functionally validated for growth (Muller et al., 2021a). The CVSC proteins UL77 and UL93 are also essential determinants of genome cleavage-packaging (DeRussy and Tandon, 2015; Borst et al., 2016). Both proteins can interact with structural capsid components, but not UL52 (Borst et al., 2016). UL77 and UL93 interaction with terminase subunits was not detected by Borst et al. (Borst et al., 2016), but has been reported by others (Köppen-Rung et al., 2016). Deletion of both proteins abrogated genome cleavage and led to accumulation of B-capsids, but no A- or C-capsids, suggesting potential structural determinants of terminase engagement (DeRussy and Tandon, 2015; Borst et al., 2016). Finally, UL34 deletion reduces viral titres by 100-fold and abrogates capsid maturation analogous to terminase mutants (Turner et al., 2022a). The precise function of UL34 is not yet clear, however RC and chromatin organisation may be involved (Turner et al., 2022a).

## Stage 8: Nuclear egress

**FIGURE 8:**
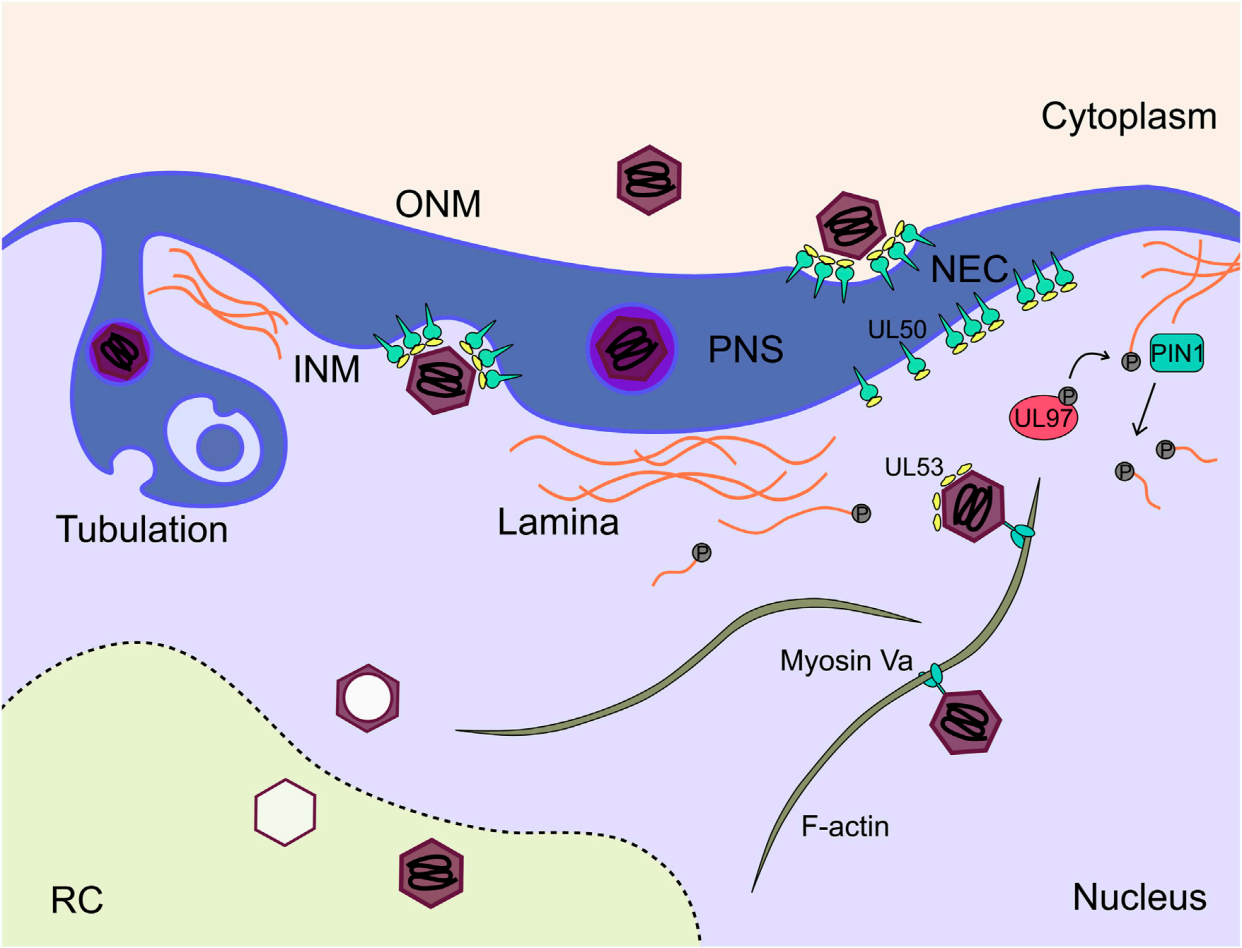
Mature capsids traverse both nuclear membranes to enter the cytoplasm for subsequent maturation. The NEC consisting of UL50 and UL53 acts as an organisational hub on the INM to recruit host and viral proteins to facilitate this step. Additionally, the nuclear lamina poses a physical barrier for exiting capsids. The lamins are phosphorylated by the viral kinase UL97 and subsequently depolymerise. C capsids travel along nuclear actin filaments to the nuclear membrane where they undergo envelopment at the INM mediated by the NEC, and subsequent fusion with the ONM to release the nascent capsid into the cytoplasm. NEC, nuclear egress complex; INM, inner nuclear membrane; ONM, outer nuclear membrane; RC, replication compartment; PIN1, peptidyl-prolyl cis-trans isomerase NIMA-interacting 1.

Maturing virions must overcome the barrier of the nuclear envelope to progress to the cytoplasm for envelopment and cellular egress. Nuclear egress proceeds through a process of envelopment at the inner nuclear membrane (INM), and de-envelopment at the outer nuclear membrane (ONM) (Figure 8). The process is broadly conserved across all herpesviruses and has been extensively reviewed (Marschall et al., 2017; Marschall et al., 2020; Arii, 2021; Draganova et al., 2021; Sanchez and Britt, 2021). The virally encoded nuclear egress complex (NEC) is composed of heterodimers of the transmembrane UL50, and soluble UL53 proteins (Dal Monte et al., 2002; Milbradt et al., 2009; Schmeiser et al., 2013; Sharma et al., 2014; Sonntag et al., 2016) with phosphorylation of both subunits required for efficient nuclear rim localisation and function (Sharma et al., 2015; Sonntag et al., 2017). Structures of herpesviral NECs have been solved (Bigalke and Heldwein, 2015; Hagen et al., 2015), including HCMV, which revealed a hook-into-groove binding interaction between the UL53 hook and UL50 groove (Leigh et al., 2015; Lye et al., 2015; Walzer et al., 2015; Muller et al., 2020). UL50-UL53 dimers oligomerise to form hexameric rings, which themselves associate as a planar lattice in the INM (Bigalke and Heldwein, 2015; Hagen et al., 2015; Leigh et al., 2015; Lye et al., 2015; Walzer et al., 2015; Muller et al., 2020). HSV-1 NECs spontaneously form vesicles *in vitro*, suggesting the NEC alone can mediate primary envelopment and membrane scission (Bigalke et al., 2014; Lorenz et al., 2015). Further, interaction of an HSV-1 CVSC (UL25/CVC2) with the NEC coat induced formation of pentameric NEC rings, and presents a potential mechanism for membrane curvature by formation of icosahedral assemblies (Draganova et al., 2020; Draganova et al., 2021). Interactions between the CVSCs and NEC subunits has been observed by some (DeRussy et al., 2016), but not others (Borst et al., 2016), and may contribute to the interactions between capsids and the NEC, as is observed for HSV-1 (Marschall et al., 2017; Sanchez and Britt, 2021). Interestingly, UL53 was observed directly on maturing capsids by immuno-gold staining (Milbradt et al., 2018), which led the authors to propose that capsid bound UL53 induces NEC lattice formation and primary envelopment (Figure 8). Recent work has validated this observation and shown that capsid bound UL53 does not influence capsid localisation to the nuclear membrane (Wilkie et al., 2022). This model is further supported by the observation that the HSV-1 homologue of UL53 recruits the UL50 homologue into complexes, but is dispensable for membrane remodelling *in vitro* (Lorenz et al., 2015). Interestingly, the NEC has selectivity for genome containing C-capsids, as few cytoplasmic B-capsids are observed (Tandon et al., 2015). For alpha herpesviruses, this selectivity has been suggested to be due to the presence of CVSCs on C-capsids (Klupp et al., 2006; Trus et al., 2007; Toropova et al., 2011; Yang et al., 2014). However, for HCMV, CVSCs were observed on all capsid types in approximately equal proportions (Borst et al., 2016). HCMV C-capsid specific structural elements including CVSC conformation, portal cap or capsid associated tegument proteins may contribute, but await investigation.

The NEC is not the sole determinant of HCMV nuclear egress, but has been proposed as a hub which recruits additional viral and cellular factors to overcome the physical nuclear barrier, and increase nuclear egress efficiency (Marschall et al., 2020). The dense nuclear lamina is a meshwork of polymerised lamins, and provides structural support to the nucleus. Whilst it facilitates nuclear organisation, phosphorylation of lamin monomers is known to cause depolymerisation and lamina breakdown (de Leeuw et al., 2018). Fascinatingly, HCMV induces lamina breakdown in areas adjacent to the vAC (Stage 6), and is mediated by the viral kinase UL97 (Marschall et al., 2005; Hamirally et al., 2009; Kuny et al., 2010; Milbradt et al., 2010). Deletion of UL97 results in an uninfected-like nuclear lamina and nuclear egress block (Krosky et al., 2003). Host kinases (Marschall et al., 2020), including protein kinase C (PKC) (Muranyi et al., 2002; Milbradt et al., 2007) have also been suggested to facilitate lamina breakdown and nuclear egress. The cellular protein p32 has been suggested to recruit UL97 to redistribute the lamina (Marschall et al., 2005). Later work showed that UL97 was recruited by the NEC but not PKC or cyclin dependent kinase 1 (CDK1) (Sharma et al., 2014). Comparison between UL97, CDK1 and PKC inhibitors revealed no effect on nuclear egress for CDK1 but a lamin-independent effect for PKC was observed (Sharma and Coen, 2014). Interestingly, UL97 has augmenting classification with deletion reducing virus titres by 100-fold (Prichard et al., 1999; Krosky et al., 2003), perhaps indicating limited redundancy in this process. Mechanistically, lamin phosphorylation is not sufficient for lamina disassembly, but instead requires the prolyl isomerase PIN1 (Milbradt et al., 2010; Milbradt et al., 2016) (Figure 8). Interaction studies have identified additional cellular proteins associated with the NEC which include p32 and emerin (Milbradt et al., 2009; Milbradt et al., 2014), with knockdown of both reducing virus production (Milbradt et al., 2014). Finally, WD repeat-containing protein 5 (WDR5) is required for efficient nuclear egress but a precise mechanism remains elusive (Yang et al., 2018).

During nuclear egress of HCMV, the precise mechanisms enabling membrane scission following primary envelopment at the INM, and de-envelopment at the ONM remain unknown. For HSV-1 and EBV, evidence has emerged that endosomal sorting complex required for transport (ESCRT) sub-units are required for efficient envelopment at the INM (Lee et al., 2012; Arii et al., 2018; Arii et al., 2022). Considering the HSV-1 NEC can form vesicles *in vitro* (Bigalke et al., 2014; Lorenz et al., 2015), ESCRT may augment the process to increase efficiency in the cellular context. For HCMV, little data exists, although no major defects in virus growth were observed in dominant negative ESCRT expressing cell lines (Streck et al., 2018). The de-envelopment process for HSV-1 and the related alphaherpesvirus pseudorabies virus (PRV), torsin A and B have been implicated (Maric et al., 2011; Hölper et al., 2020), as well as the envelope glycoprotein gB for HSV-1 (Farnsworth et al., 2007; Wright et al., 2009). Interestingly, PRV has no dependence on gB for de-envelopment and this suggests mechanistic differences within the same subfamily (Schulz et al., 2013), precluding other assumptions based on related virus species. For nuclear de-envelopment of HCMV, no specific studies have yet been reported.

Maturing capsids must travel a significant distance from the site of capsid assembly and genome packaging at the RC periphery (Stage 7), to the cytoplasmic vAC (Stage 6) for secondary envelopment and egress (Stages 9–10). The LINC complex connects cytoplasmic microtubules to the nucleoskeleton [reviewed (Starr and Fridolfsson, 2010; Simon and Wilson, 2011)]. Procter et al. have recently demonstrated that the LINC is integral to both nuclear and cytoplasmic organisation during HCMV infection including nuclear F-actin rearrangement and chromatin localisation (Procter et al., 2020). F-actin filaments are induced by HCMV infection and form along the RC periphery and extended to the nuclear rim (Wilkie et al., 2016). Inhibition blocked virus production, cytoplasmic capsid accumulation and localisation of nuclear capsids to the nuclear rim (Wilkie et al., 2016). Follow-up work showed the motor protein myosin Va colocalised with F-actin and capsids, with knock-down resulting in similar capsid localisation and nuclear egress defects (Wilkie et al., 2018) (Figure 8). In addition to lamina breakdown at the INM adjacent to the vAC (Marschall et al., 2005; Hamirally et al., 2009; Kuny et al., 2010; Milbradt et al., 2010), the perinuclear space enlarges during infection and increases in permeability, with binding immunoglobulin protein (BiP) and possibly SUN domain proteins involved (Buchkovich et al., 2010; Klupp et al., 2017). 3D tomography of the nuclear membrane during infection further reinforces the notion that the nuclear membrane is dramatically remodelled (Villinger et al., 2015). Large infoldings of the INM were typical, with perinuclear virions and vesicles observed within these (Figure 8). Strikingly, second and third order infoldings were also observed consistent with a large expansion of the INM and a “pushing membrane” model of capsid envelopment (Villinger et al., 2015). Taken together, the nuclear membrane is remodelled during infection and the F-actin-LINC-microtubule continuum influences nuclear morphology and traffic of maturing capsids from the RC to the cytoplasm. These observations will inform future mechanistic investigations of HCMV nuclear egress.

## Stage 9: Envelopment of maturing nucleocapsid

**FIGURE 9:**
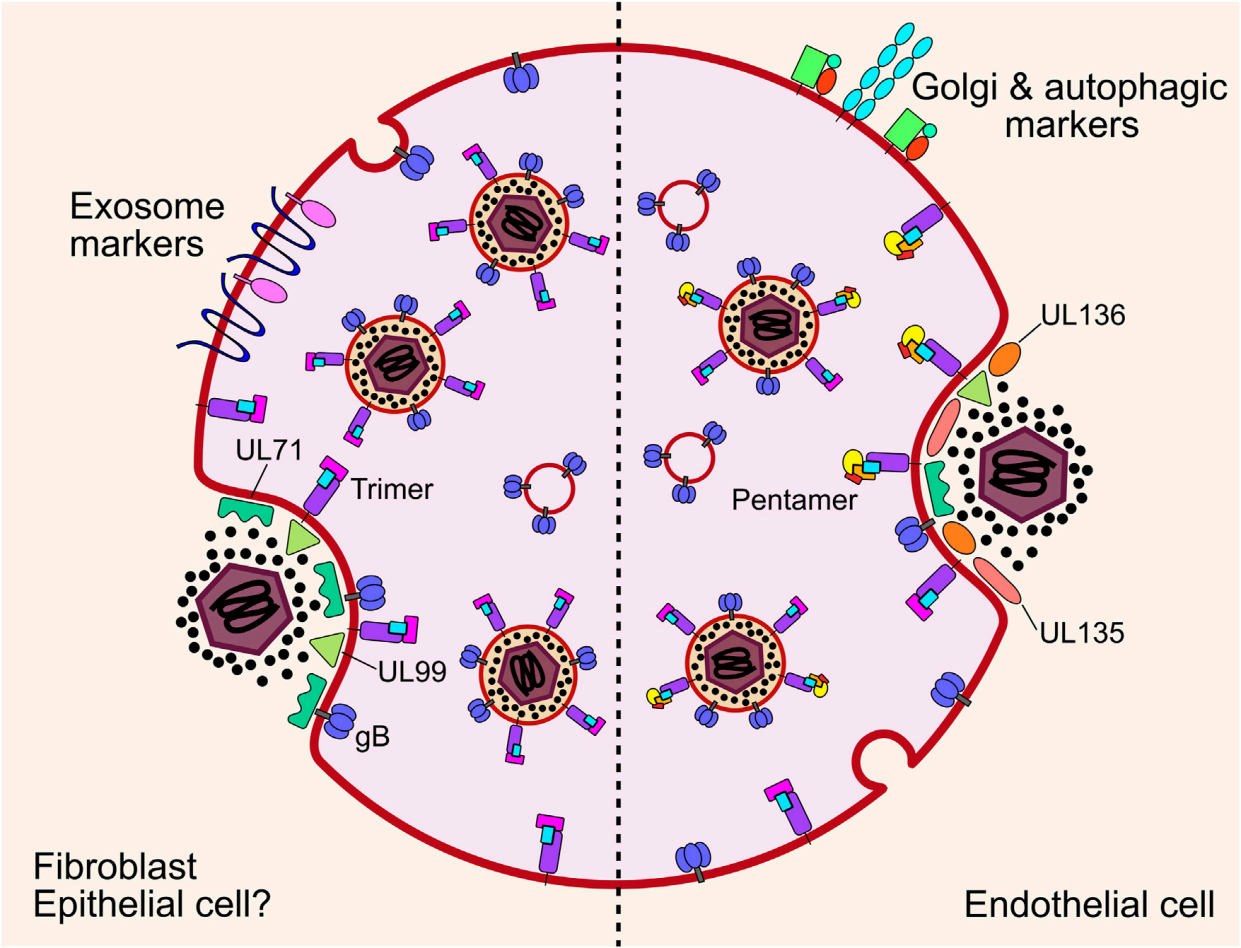
Cytoplasmic envelopment of nascent virions occurs on MVB membranes or short cisternae within the vAC. In fibroblasts and likely epithelial cells, envelopment is mediated by multiple envelope glycoproteins and the membrane associated tegument proteins UL71 and UL99. The site of final envelopment as well as the virion envelope are enriched in exosome markers that indicates a common membrane origin. In endothelial cells, final envelopment also occurs on MVB membranes. However, these are not enriched in exosome markers, but rather contain Golgi and autophagic markers. Additionally, the viral proteins UL135 and UL136 are important for envelopment in endothelial cells, but are entirely dispensable in fibroblasts. Note: UL71 and UL99 are included in the diagram for endothelial cell envelopment, as they are well conserved in clinical strains. However, they have not been directly assayed in endothelial cells. MVB, multivesicular body; gB, envelope glycoprotein B.

Key evidence supporting the vAC as the final site for virion envelopment comes from the localisation of viral envelope glycoproteins gL, gH and gB. These were shown to co-localise with various markers of the *trans*-Golgi network (TGN), endocytic, and secretory pathways within the central areas of the vAC (Homman-Loudiyi et al., 2003). Interestingly, many of these markers were also detected in infected cell culture supernatant and purified virions including CD63, TGN46, transferrin receptor (TFRC) and cation-independent mannose-6-phosphate receptor (CI-M6PR/IGFR) (Cepeda et al., 2010). Based on these observations, a model whereby HCMV generates a hybrid membrane compartment at the centre of the vAC exhibiting characteristics of both endosomal and TGN membranes for virion envelopment was proposed (Cepeda et al., 2010). Building on this, Schauflinger et al. (Schauflinger et al., 2013) generated 3D models of the HCMV vAC using cryogenic electron microscopy sections. This revealed nucleocapsids accumulate in a zone bounded by the Golgi ring. Furthermore, single virions were observed budding into short cisternae as well as multiple enveloped virions inside a single multivesicular body (MVB). In some instances, enveloped virions were observed alongside intraluminal vesicles (ILV), in a single MVB, which has been observed by others (Fraile-Ramos et al., 2002; Fraile-Ramos et al., 2007; Schauflinger et al., 2011) (Figure 9). The late endosomal marker CD63 was also detected in HHV6 virion envelopes as well as adjacent ILVs by immuno-gold labelling (Mori et al., 2008), suggesting MVB envelopment is conserved across the betaherpes subfamily. Rather than generating entirely new cellular processes, HCMV is known to beneficially hijack existing host pathways (Alwine, 2012), and interestingly, the MVB pathway is inexorably tied to exosome biogenesis.

Exosomes are membranous nanovesicles 30–200 nm in size and secreted from all cell types. Inward budding of endosomal membranes form ILVs within a limiting endosomal membrane to form an MVB. MVBs can fuse with lysosomes resulting in cargo degradation, or they can traffic to the plasma membrane to release the ILVs into the extracellular space as exosomes [reviewed (Raposo and Stoorvogel, 2013; Colombo et al., 2014; Hessvik and Llorente, 2018)]. Exosomes have emerged as important players in a suit of normal and pathological processes, including cell-cell communication and metastatic niche formation (Weidle et al., 2017). Isolation and proteomic profiling of uninfected cell exosomes, HCMV infected cell exosomes (viral exosomes) and virions revealed complete incorporation of the exosome proteome into mature virions, further suggesting a common membrane origin for both populations (Turner et al., 2020). Analysis of the viral protein composition of viral exosomes revealed enrichment of envelope glycoproteins (Zicari et al., 2018; Streck et al., 2020; Turner et al., 2020; Bergamelli et al., 2022), including UL132 (Turner et al., 2020; Wu et al., 2020), as well as outer tegument proteins involved in envelopment (Turner et al., 2020), further supporting the intrinsic nature of the exosome pathway in the egress continuum. Complementing these findings, recent work using 3D CLEM showed unequivocally that HCMV virions and other vesicles bud into and accumulate in MVBs positive for markers of the endocytic trafficking system and the exosome pathway (Flomm et al., 2022). Compilation and re-analysis of multiple HCMV and extracellular vesicle proteomes has revealed signatures that suggest envelopment occurs on membranes derived from tubular recycling endosomes (Mahmutefendić Lučin et al., 2022). Intriguingly, comparison of envelopment between fibroblasts and ECs revealed divergence in cellular markers on MVB limiting membranes (Momtaz et al., 2021). In both cell types, virions and ILVs were observed in common MVBs, however, host markers of the limiting membranes differed. Endocytic and exosomal membranes associated with fibroblast MVBs, while golgi and autophagic markers were present for ECs (Momtaz et al., 2021) (Figure 9). Autophagic membranes have also been suggested as sites of HCMV envelopment in fibroblasts (Taisne et al., 2019), however, these proteins had low virion enrichment compared to other host proteins (Turner et al., 2020), and moderate fold changes in virion production when depleted (Taisne et al., 2019). Interestingly, secretory autophagy is known to produce extracellular vesicles (Ponpuak et al., 2015; Pleet et al., 2018) and may be co-opted by HCMV for envelopment in some contexts.

HCMV envelopment has proven to be more complex than HSV-1, with the involvement of ESCRT components remaining controversial. Consistent with HSV-1, tumor susceptibility gene 101 (TSG101) and ALG-2-interacting protein X (ALIX) were reported to be dispensable for HCMV production (Fraile-Ramos et al., 2007; Tandon et al., 2009), however, the ESCRT-III complex and vacuolar protein sorting 4 (VPS4) phenotypes appear to be somewhat dependent upon the inhibition system and virus strain used. Fraile-Ramos et al. (Fraile-Ramos et al., 2007) demonstrated that transient knockdown of VPS4 using siRNA increased virus production and concluded that VPS4 was dispensable for HCMV envelopment. This was subsequently refuted in a follow-up study by Tandon et al. (Tandon et al., 2009) who employed a dominant-negative system to inhibit ESCRT-III component Charged multivesicular body protein 1 (CHMP1) and VPS4, and observed a significant reduction in production of infectious virus in cell lines overexpressing both dominant negative CHMP1 and VPS4 by cell-to-cell spread. The former study was criticised for the incompatibility between the fibroblast specific AD169 virus strain and retinal pigment epithelial cells (RPE-1) used (McSharry et al., 2001). The most recent work to investigate ESCRT-III and VPS4 involvement in HCMV envelopment utilized stable cell lines with inducible expression of dominant negative forms of the protein to control for any cellular toxicity (Streck et al., 2018). All ESCRT components and VPS4 were dispensable for extracellular virus production, but a modest defect in cell-to-cell spread was observed, in partial agreement with Tandon et al. (Tandon et al., 2009). With the emergence of ESCRT-independent ILV formation (Hessvik and Llorente, 2018), HCMV may hijack these processes, a hypothesis congruent with low ESCRT enrichment observed in HCMV virions and exosomes from infected cells (Turner et al., 2020). Virion envelopment is known to be dependent on multiple viral proteins, and a more definitive understanding of the contribution from host proteins is ongoing.

From the viral perspective, the tegument protein UL99 localises to the centre of the vAC during infection (Sanchez et al., 2000a), and is known to be essential for virion production (Silva et al., 2003). An acidic cluster of amino acids at the N-terminus is essential for UL99 traffic to the vAC (Seo and Britt, 2006), as is phosphorylation (Seo et al., 2020). UL94 and M94 are essential for secondary envelopment (Maninger et al., 2011; Phillips and Bresnahan, 2012), with interactions between UL99 and UL94 required (Phillips et al., 2012). UL71 is also involved in envelopment by several independent groups using viruses lacking UL71 (Womack and Shenk, 2010; Schauflinger et al., 2011). In the absence of UL71, partially enveloped virions aggregated around the periphery of enlarged MVBs, and an overall reduction in viral titre was observed by both. An N-terminal tyrosine trafficking motif is required for UL71 localization to the vAC (Dietz et al., 2018), and mutation of a C-terminal tetralysine motif resulted in similar envelopment defects to complete UL71 deletion (Read et al., 2019) (Figure 9). An egress defect post envelopment was suggested for UL103, however a precise mechanism was not reported (Ahlqvist and Mocarski, 2011). The HSV-1 homologue of UL103 (UL7) forms a complex with the UL71 homologue (UL51) to mediate envelopment (Butt et al., 2020). Interestingly, structural homology between HSV-1 UL51 and ESCRT-III subunits was revealed, which suggests herpesviruses encode protein complexes to bypass ESCRT mediated budding, and perhaps scission (Butt et al., 2020). The envelope glycoproteins also appear to be an important determinant of envelopment, with gM and gN forming a stable complex through disulphide bonds (Mach et al., 2000; Mach et al., 2005). The cytoplasmic tail of gM has a trafficking motif required for vAC localisation of gM/gN, with deletion inhibiting virion maturation (Krzyzaniak et al., 2007). The cytoplasmic domain of gN contains essential cysteine residues, with substitution mutations causing a likely defect in envelopment (Mach et al., 2007). Additionally, a contribution from gO to secondary envelopment in fibroblasts and ECs has been demonstrated (Jiang et al., 2008).

The divergent MVB membrane composition between fibroblasts and ECs is further illustrated by the requirement of the UL133/8 locus for efficient tegumentation and envelopment in ECs, but not fibroblasts (Bughio et al., 2013). In ECs, deletion of UL135 abrogates envelopment at the MVB membrane and UL136 mutants display enlarged dense bodies (Bughio et al., 2015) (Figure 9). Likewise, deletion of the UL133/8 locus alters vAC morphology in ECs (Bughio et al., 2013). Deletion of UL133/8 is completely dispensable for envelopment and viral growth in fibroblasts when deleted from the endotheliotropic TB40E or FIX strains (Bughio et al., 2013), while the locus is entirely lost from fibroblast passaged AD169 and Towne (Cha et al., 1996; Murphy et al., 2003). Interestingly, robust viral titres were recovered from epithelial cells infected with AD169 when susceptibility was restored either by repairing the pentamer (Wang and Shenk, 2005; Adler et al., 2006), or overexpression of the trimer ligand PDGFRα on the target cell (Vanarsdall et al., 2012; Wu et al., 2018), which strongly suggests epithelial cells are permissive for replication after successful entry. Similar results were also observed for ECs infected with AD169 with repaired pentamer, although absolute titres were much lower (Wang and Shenk, 2005; Adler et al., 2006), but nonetheless diverges from the UL133/8 deletion results (Bughio et al., 2013; Bughio et al., 2015). The different MOIs, time points and viral strains used between studies complicates direct comparisons which awaits confirmation. Additionally, it has been observed that different cell types release distinct virus populations with altered tropism and envelope composition (Scrivano et al., 2011). Cell-type specific mechanisms of virion cargo recruitment and envelopment likely influences viral dissemination *in vivo*, with wider implications for clinical management of HCMV.

## Stage 10: Virion egress and release

**FIGURE 10:**
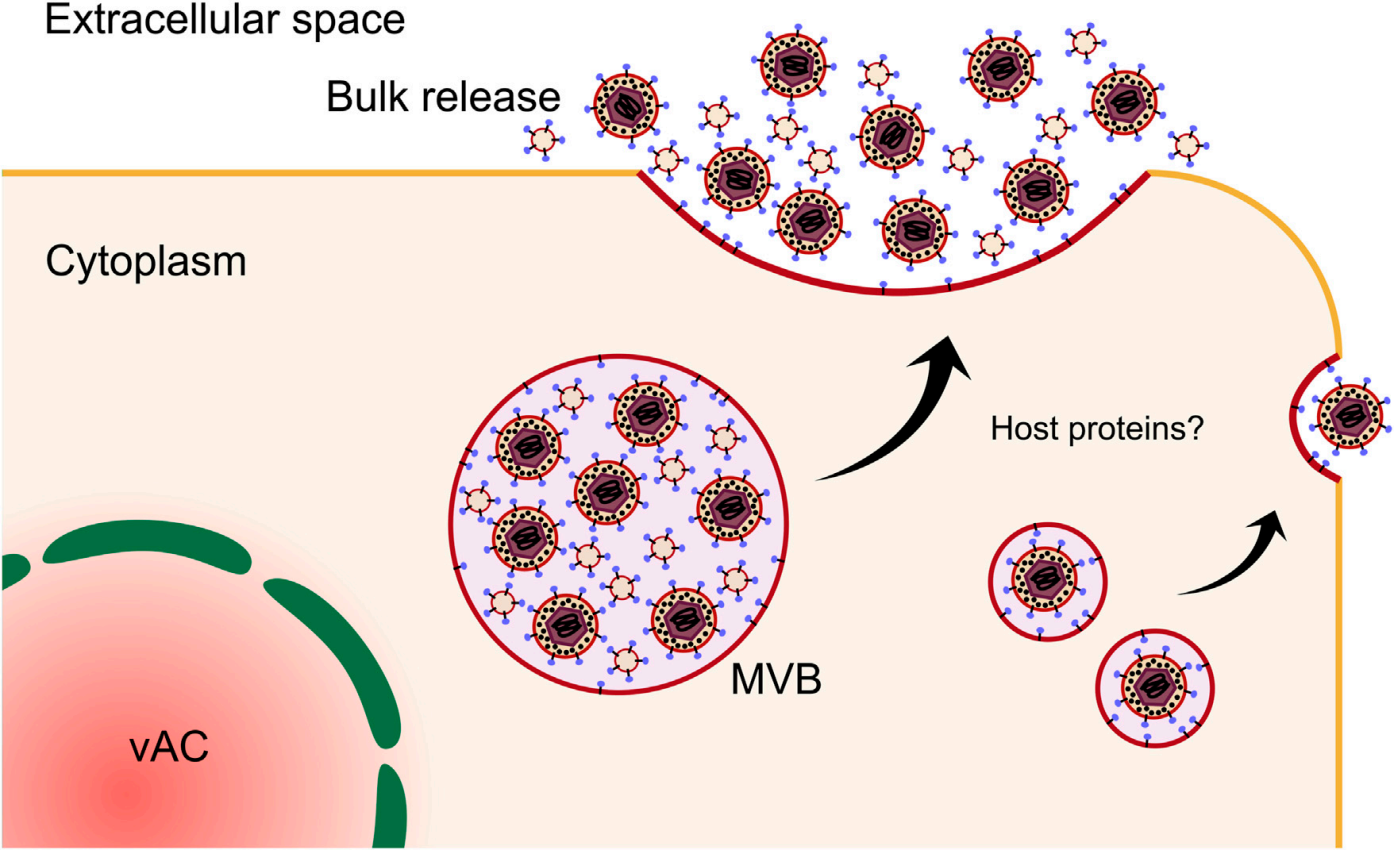
Following envelopment, MVBs containing enveloped virions and ILVs traffic to the PM, fuse, and release virions and exosomes into the extracellular space through a bulk release mechanism. Simultaneously, virions that undergo envelopment on individual vesicles are thought to fuse directly with the PM to release single virions. Few regulators of virion egress have been characterised to date, however, many host proteins linked to exosome secretion have been associated with viral growth with involvement in egress. Cell type specific divergence, as well as cell-to-cell spread mechanisms, may exist in contrast to the schematic outlined. MVB, multivesicular body; vAC, viral assembly compartment.

After secondary envelopment, MVBs containing ILVs and virions traffic to the plasma membrane and undergo fusion to release infectious progeny extracellularly. Early observations of virion egress using fluorescently labelled capsids indicated it to be a random and inefficient process. Capsids showed small irregular movements with no clear direction, yielding approximately 1 infectious virion per cell per hour in culture (Sampaio et al., 2005). More recent investigations have reported that egress occurs intermittently, with bulk release of virions and vesicles at the PM following MVB fusion (Flomm et al., 2022) (Figure 10). Additionally, given the differences in envelopment mechanisms for ECs versus fibroblasts, egress mechanisms in different cell types may diverge or have redundancies depending on the cellular context (Wedemann et al., 2022). Illustrating this, pentamer expression appears to influence not just tropism, but also extracellular versus cell-to-cell spread. AD169 with repaired pentamer resulted in reduced extracellular viral titres in fibroblasts compared to the parental virus, highlighting a possible assembly, envelopment, or egress contribution (Wang and Shenk, 2005; Adler et al., 2006). Whether pentamer dependent effects on viral envelopment and/or egress are fitness enhancing *in vivo*, has not yet been established.

Molecular and mechanistic knowledge of cellular egress in general is lacking, however, some proteins have been identified. Interestingly, cellular egress appears to be primarily driven by host proteins, and many of these are implicated in exosome biogenesis and secretion, featuring Rab GTPases and SNAREs. RAB27A localised to virion membranes and the vAC, and knock-down reduced virus titre by approximately 3-fold (Fraile-Ramos et al., 2010). Whilst this is a modest reduction, RAB27A inhibition reduced exosome production by only 2-fold (Ostrowski et al., 2010), and similar undiscovered redundancies may exist as for exosome secretion. Inhibition of RAB4B (McCormick et al., 2018) and RAB11 (Krzyzaniak et al., 2009) also reduced viral titres, as did STX3 (Cepeda and Fraile-Ramos, 2011), with synaptosome associated protein 23 (SNAP23) inhibition reducing virus titres by approximately 1000-fold (Liu et al., 2011). Little mechanistic work has been performed to pinpoint these exact defects, with likely function inferred from known functions in the cell in the absence of infection. Given the complete remodelling of the vAC and MVB membranes during infection, pre-envelopment dependencies cannot be excluded for all host factors mentioned.

## Discussion

**FIGURE 11:**
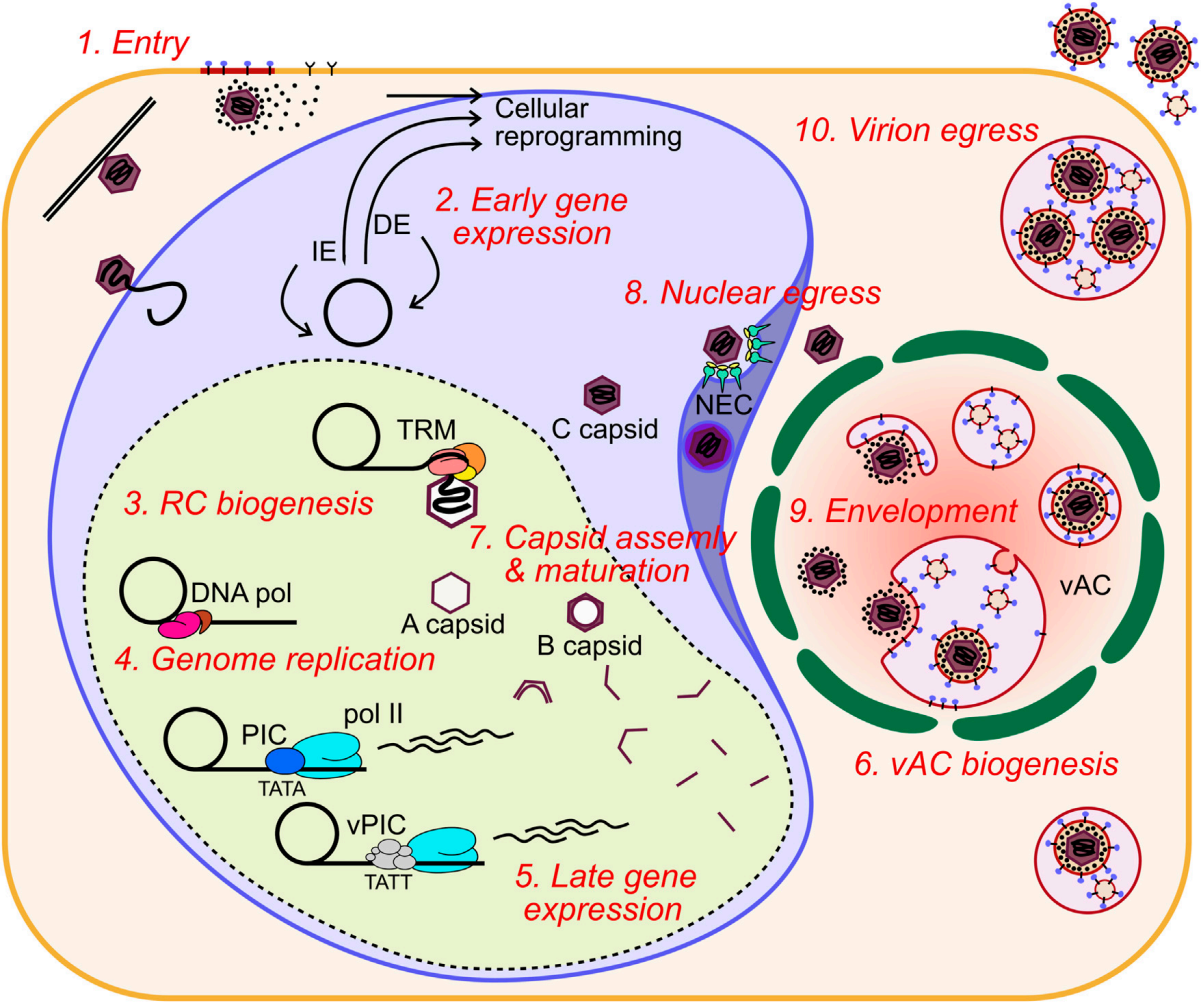
HCMV lytic replication proceeds sequentially through 10 checkpoints, with completion of each essential for production of infectious virions. These are 1) entry, 2) early gene expression, 3) RC biogenesis, 4) viral genome replication, 5) late gene expression, 6) vAC biogenesis, 7) capsid assembly and maturation, 8) nuclear egress, 9) envelopment, and 10) cellular egress. IE, immediate early; DE, delayed early; DNA pol, viral DNA polymerase complex; RC, replication compartment;, PIC, pre-initiation complex; vPIC, viral pre-initiation complex; pol II, RNA polymerase II complex; TRM, tripartite terminase complex; NEC, nuclear egress complex; vAC, viral assembly compartment.

Viral replication is a remarkable logistical feat that occurs at the molecular level, with both host and viral processes underpinning various stages along the replication continuum. We described 10 stages in total: 8 essential viral replication steps and the biogenesis of 2 virally-induced cellular megastructures (RCs and vAC). Based on the literature to date, and our own observations, the idea emerging is that progression to the following stage is dependent on initiation of the previous (Figure 11). We appreciate that our model may be somewhat simplified as this biological system is incredibly complex, and there may be concurrent processes, feedback loops and cross-talk at various stages which are not yet appreciated. Nonetheless, the sequential nature of virion maturation, and the linked dependency provides multiple cellular loci to halt the entire replication process and provides a useful framework to investigate defects in viral replication progression. Each stage represents an inherent vulnerability for future antiviral development, with multiple specific targets likely within each. It is clear that some replication stages are understood in more detail than others. For example, our knowledge of cellular entry (Stage 1) and nuclear egress (Stage 8) is greater than for late viral transcription mechanisms (Stage 5), capsid assembly (Stage 7), envelopment (Stage 9), and virion egress (Stage 10). From our perspective, understanding the mechanistic details of these events will deliver potential for the development of future therapeutics.

Late viral gene transcription, exemplified by the betagamma vPIC, but also including IE2 dependent mechanisms (Stage 5) (Figure 5), represent a distinct checkpoint in the replication cycle (Figure 11) with limited antiviral development. The vPIC specifically binds viral promoters and recruits host RNA pol II for viral gene transcription. Based on studies in both beta- and gamma-herpesviruses, it is thought that UL87 and its analogues substitute for host TBP in the PIC (Wyrwicz and Rychlewski, 2007; Gruffat et al., 2012; Davis et al., 2015; Gruffat et al., 2016; Castañeda et al., 2020). The vPIC alone is an attractive antiviral target as there are 6 sub-units in the complex, all of which are essential for replication, which likely requires a constellation of compatible mutations to yield a resistant mutant that retains fitness. Given the vPIC substitutes for components of the host PIC, an alternative inhibition strategy could be designed to target the vPIC-pol II interface providing the selectivity and tolerability of traditional antivirals while also blocking pol II function in the host-viral complex, and thus reduce the emergence of drug-resistant mutant viruses further. Regardless of the exact inhibition strategy, a vPIC specific inhibitor would offer an additional alternative to existing licensed drugs and be compatible in combination. Future mechanistic work defining the functional domains, interaction interfaces, and complete structures of the vPIC and vPIC-Pol II complex is therefore of significant clinical interest.

While pro-capsids can self-assemble *in vitro*, complete capsid maturation to form DNA-filled C-capsids is more complex and requires additional steps. These include capsid angularisation, scaffold cleavage and removal, addition of CVSCs, binding of capsid-associated tegument proteins (e.g., UL32), terminase engagement with the portal complex, genome translocation, genome cleavage, and portal capping [reviewed (Heming et al., 2017; Muller et al., 2021b)]. The order and concurrent progression are not currently known for any herpesvirus, but some interdependencies can be inferred. When the HCMV maturational protease is inhibited, angularised capsids superficially similar to B-capsids form (Yu et al., 2005). Protease null capsids display filamentous connections between the scaffold ring and the capsid floor, indicating that cleavage of the scaffold c-terminal domain is not essential for capsid angularisation (Yu et al., 2005), but is probably required for scaffold removal. Additionally, protease null capsids have decreased density inside the scaffold ring which suggests much of the protease resides there, even after cleavage of the c-terminal scaffold domain (Yu et al., 2005). Cleaved scaffold is removed from the capsid before DNA is packaged, but how this is initiated and how it proceeds is not known. It has been suggested that cleaved scaffold is extruded through channels in the pro-capsid shell before or during angularisation, based on structural comparison between pro-capsids and B-capsids in HSV-1 (Brown and Newcomb, 2011), but there is no direct evidence. While there remains much to uncover in terms of mechanisms, what is clear is that maturational protease executes an essential function and represents an excellent target for antiviral development. Viral protease inhibition using small molecules has precedent (Borthwick, 2005), and is further highlighted by HIV and HCV protease inhibitors in widespread clinical use (Kurt Yilmaz et al., 2016).

Envelopment of HCMV capsids is intimately entwined with host cell architecture and membrane organisation. Assignment of a precise cellular origin of the virion envelope is complicated by the dramatic remodelling of host cellular organelles during HCMV infection which masks their former identity (Jean Beltran et al., 2016). Profiling virion proteomes and cataloguing cargo, combined with functional validation assays offers an unbiased approach to describe the cellular envelope origin and uncover important mechanisms. It is emerging that the ESCRT complexes and associated VPS4 are non-essential for HCMV replication (Fraile-Ramos et al., 2007; Tandon et al., 2009; Streck et al., 2018). Therefore, there is no definitive evidence of any host proteins that mediate envelopment of HCMV virions, but future work may yet uncover examples. Also, yet to be defined host processes likely facilitate trafficking of essential viral proteins to the site of envelopment, as well as shape MVB microdomains essential for envelopment. Evidence of divergent MVB membrane composition between fibroblasts and ECs (Momtaz et al., 2021), bolstered by the requirement for the UL133/8 locus in these cells (Bughio et al., 2013; Bughio et al., 2015), adds further complexity to these processes, as does the effect of pentamer expression on virus titres in fibroblasts, which appears to modulate envelopment or egress (Wang and Shenk, 2005; Adler et al., 2006). To overcome these hurdles and characterise the cell type specific envelopment pathways, and the viral dependencies of each, multiple virus strains and cell types will need to be utilised in side-by-side assays. Additionally, introducing deletion and repair mutations (e.g., UL128-UL131A and UL133/8 loci) in each strain will provide valuable mechanistic insights. For efficient dissemination, HCMV targets specific cell types in an orchestrated manner (Jackson and Sparer, 2018). Assembling distinct populations of virions in different cell types with varied virion composition and tropisms is one viral strategy which has experimental support (Scrivano et al., 2011), and offers potential explanations for the divergent envelopment and egress pathways between cell types. The core viral proteins UL71, UL94, UL99 and UL103 have all been reported to localise to the vAC and support envelopment of cytoplasmic virions, with UL71 the most well characterised (Womack and Shenk, 2010; Schauflinger et al., 2011; Dietz et al., 2018). Considering recent work, it is likely UL71 mediates membrane budding analogously to ESCRT-III, as its HSV-1 homologue oligomerises to form filaments with structural homology to these subunits (Streck et al., 2018; Butt et al., 2020). Precisely how membrane scission is executed, and whether it is mediated by host or viral proteins, or both, remains an open question. Mechanistic detail of how these host and viral proteins function and interact is not yet established. The opportunity to study these processes in more depth is emerging, with advances in technology, notably super resolution fluorescent microscopy. Characterisation of molecular mechanisms governing envelopment will reveal essential enzymatic activity or interaction interfaces during this replication stage. Furthermore, delineating cell type specific envelopment and egress mechanisms, and the influence on virion composition and tropism, will be valuable for the clinical management of HCMV.

Cytoplasmic egress, and the associated cellular mechanisms, is by far the least understood stage of the replication cycle. What we can conclude is that host cell vesicular trafficking proteins are of high interest, including the Rab GTPases and SNAREs (Stage 10). Viral proteins may perform essential virion egress functions, but none have yet been definitively demonstrated. Defining processes occurring during the final stage of the replication cycle is particularly difficult given the potential for non-specific, or toxic cellular effects stalling viral progression at earlier replication stages and confounding the results. Method development to overcome these challenges will be of immense value. For example, optimising inducible cellular promoters or degradation tag systems in permissive cell types to study host protein function late in infection will help to define the critical host and viral determinants of virion egress, and unlock novel antiviral targets.

The development pipeline for new HCMV antivirals is constantly evolving as established candidates fail and new compounds are discovered or repurposed. The licensed drugs to date, as well as multiple related derivatives, target UL54 (Stage 4), the terminase (Stage 7), and UL97 (Stage 8) (Table 1) (Britt and Prichard, 2018). Prospective small molecule inhibitors have recently been reviewed by others and overwhelmingly target the previously mentioned proteins together with viral entry (Table 1) (Bogner et al., 2021). Viral inhibition through active and passive immunisation is a broad area of investigation and beyond the scope of this review and has been covered in detail by others (Plotkin et al., 2020; Griffiths and Reeves, 2021). The complex herpesvirus replication cycle offers diverse and numerous replication stages for restriction which have been outlined in this review. At each replication stage there exist multiple opportunities for restriction (Table 1), with more to emerge in the future as additional mechanisms are characterised. In our opinion, inhibitors of the helicase-primase complex (Stage 4), betagamma vPIC (Stage 5), maturational protease/UL80a (Stage 7), NEC (Stage 8) and UL71 (Stage 9) offer the most opportunity to screen for inhibitors based on current knowledge (Table 1). There is still much to learn about the fundamental mechanisms of HCMV replication which will illuminate new candidates for restriction along the replication continuum.

**TABLE 1:** Summary of approved HCMV antivirals and putative targets for future antiviral development.

Stage	Approved drug (s)	Target (s)	Putative target (s)
1: Viral entry into host cells			Trimer, pentamer, gB
2: Nuclear trafficking and early viral gene expression			IE1
3: Establishment of the nuclear replication compartment			UL112/UL113
4: Genome replication	Ganciclovir, Valganciclovir, Cidofovir, Foscarnet	UL54	DNA pol (UL44, UL54), helicase-primase (UL70, UL102, UL105)
5: Late gene expression			vPIC (UL49, UL79, UL87, UL91, UL92,UL95), IE2, RNA pol II
6: Biogenesis of the viral assembly compartment			UL132
7: Capsid assembly and genome packaging	Letermovir	UL56, UL89	Maturational protease (UL80a), terminase (UL51, UL56, UL89), UL52, UL77, UL93
8: Nuclear egress	Maribavir	UL97	NEC (UL50, UL53), UL97, PIN1, LINC, F-actin, MYO Va
9: Envelopment of maturing nucleocapsid			UL71, UL94, UL99, UL103?
10: Virion egress and release			Rab GTPases? SNAREs?

Broad spectrum or pan herpes antiviral molecules could also offer clinical benefit as multiple herpesvirus species can reactivate simultaneously following transplantation (Dzieciatkowski et al., 2016; Quintela et al., 2016; Anderson-Smits et al., 2020). This is not an intractable proposition as core herpesvirus genes drive multiple stages of the replication cycle and are largely conserved across subfamilies. DNA replication (Stage 4), capsid assembly and genome packaging (Stage 7), and nuclear egress (Stage 8) share many similar mechanisms across subfamilies, which in our view, currently offer the most promising opportunities to pursue this approach. As more details emerge, it is likely that additional molecular mechanisms will converge across the herpesviruses and reveal development opportunities for broad spectrum inhibitors.

For successful completion of the replication cycle, HCMV must progress through each of the 10 essential stages outlined in this review. For each event there is a contribution from both host and viral factors, and targeting this nexus may help to limit antiviral mutation. Research to date has at least partially characterised the majority of the essential HCMV genes, with some critical structures such as envelope glycoproteins, the capsid and NEC having complete structures solved. For others, mechanisms are still lacking, with the late cytoplasmic stages encompassing virion envelopment and egress the most poorly understood. From the host perspective, only a handful of essential proteins have been identified, and the depth of mechanistic knowledge can be improved. In the future, large scale screening approaches to identify all host and viral determinants of each replication stage will be valuable if technical limitations can be overcome, and appropriate assays designed. This will pave the way for development of new drugs and therapies inhibiting not just the viral DNA polymerase, but also multiple steps along the replication continuum. Effective combination therapy will guard against the emergence of future resistant strains, and preserve the efficacy of existing drugs.
